# Disease‐Stage Synchronized Nanozyme Therapy for Polymicrobial Sepsis through Adaptive Catalytic and Immune Reprogramming

**DOI:** 10.1002/advs.77231

**Published:** 2026-09-08

**Authors:** Anlai Zou, Xiaoxue Zhu, Runhan Zhu, Chengchu Xue, Qinyu Zhang, Mengke Li, Ting Yu, Yuzhen Gao, Zhi Ruan, Ying Fu, Yunlei Xianyu, Jun Zhang

**Affiliations:** ^1^ Department of Clinical Laboratory Sir Run Run Shaw Hospital of Zhejiang University School of Medicine Hangzhou Zhejiang China; ^2^ Key Laboratory of Precision Medicine in Diagnosis and Monitoring Research of Zhejiang Province Hangzhou Zhejiang China; ^3^ College of Biosystems Engineering and Food Science Zhejiang Key Laboratory of Agro‐Food Resources and High‐Value Utilization Zhejiang University Hangzhou China

**Keywords:** anti‐inflammation, cuproptosis, immunomodulation, pH‐adaptive, sepsis

## Abstract

Sepsis remains a leading cause of mortality because current therapies fail to address its dynamically evolving pathophysiology, in which infection, oxidative stress, and immune dysfunction emerge sequentially and interdependently. Here, we present a pH‐adaptive nanozyme platform (MICP@HG) that orchestrates stage‐specific antibacterial and immunomodulatory activities throughout sepsis progression. The platform integrates near‐infrared imaging, catalytic therapy, and immune regulation into a single construct. In acidic infectious microenvironments, the Cu–piceatannol shell exhibits peroxidase‐mimicking activity and induces cuproptosis‐like bacterial death through metabolic collapse and redox imbalance. As the microenvironment normalizes, the nanozyme shifts toward antioxidative and anti‐inflammatory functions via superoxide dismutase (SOD)‐ and catalase (CAT)‐like activities. Concurrently, the hyaluronic acid (HA)/β‐glucan coating facilitates infection‐targeted delivery and reprograms macrophages toward a reparative phenotype while restoring immune responsiveness. This dynamic functional transition enables efficient eradication of multidrug‐resistant bacteria, attenuation of systemic inflammation, and preservation of organ function, ultimately achieving complete survival in polymicrobial sepsis models. Notably, the platform also elicits a vaccine‐like trained immunity effect that confers protection against reinfection. This work establishes a paradigm for temporally programmed nanotherapy that aligns therapeutic function with disease progression, offering a precision strategy for the treatment of complex inflammatory disorders.

## Graphical abstract

A disease‐stage synchronized nanozyme platform is developed to treat polymicrobial sepsis by adapting its functions to changing infection environments. The system eliminates multidrug‐resistant bacteria during early infection while reducing inflammation and restoring immune balance during recovery, providing an integrated strategy for infection control and host protection.

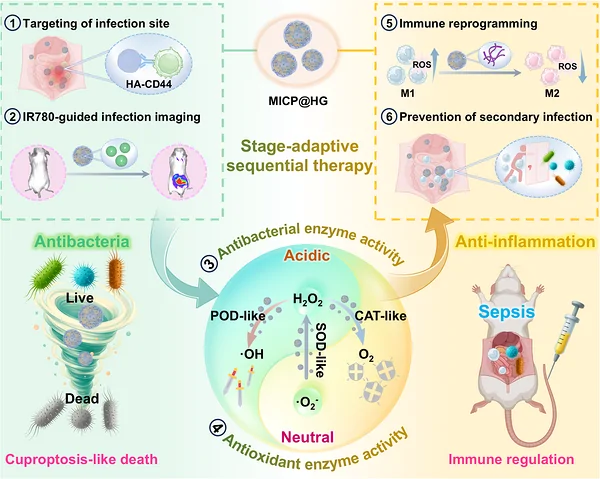

## 1 Introduction

Sepsis is a life‐threatening systemic syndrome characterized by a dysregulated host response to infection that frequently progresses to multiorgan failure and remains associated with high mortality worldwide [1, 2, 3, 4, 5]. Despite advances in antimicrobial therapy and supportive care, clinical outcomes remain poor, with mortality rates exceeding 40% in critically ill patients. A major obstacle to effective treatment lies in the highly dynamic pathophysiology of sepsis, which evolves from early pathogen proliferation to uncontrolled inflammation, oxidative injury, and ultimately immunosuppression [6, 7, 8, 9, 10]. However, current therapeutic strategies remain largely static and reductionist, relying on either broad‐spectrum antibiotics or anti‐inflammatory interventions that fail to accommodate the temporally evolving interplay among infection, redox dysregulation, and immune dysfunction [8, 11, 12]. This mismatch between therapeutic modality and disease stage not only limits therapeutic efficacy but also contributes to therapeutic failure and disease recurrence.

Recent advances in multifunctional nanomaterials have sought to integrate antibacterial and anti‐inflammatory capabilities, yet these systems generally operate in a constitutive and non‐adaptive manner, thereby lacking the capacity to dynamically coordinate therapeutic functions in response to disease progression [13, 14]. As a result, they fail to reconcile the opposing demands of early‐stage bactericidal activity and late‐stage cytoprotection and immune restoration. This limitation highlights a critical unmet need for temporally programmable therapeutic systems capable of adapting to the evolving microenvironment of sepsis. Notably, the microenvironment of septic lesions undergoes pronounced physicochemical changes during disease progression [15]. Infected tissues typically exhibit an acidic microenvironment (pH 4.5‐6.5) due to hypoxia, lactic acid accumulation, and inflammatory infiltration, which gradually returns toward physiological neutrality during inflammation resolution [16, 17, 18, 19]. This dynamic pH evolution provides an opportunity to design stimuli‐responsive nanoplatforms capable of temporally regulating reactive oxygen species (ROS) homeostasis and therapeutic activity. We therefore hypothesized that a pH‐adaptive system with multienzyme‐like activities could enable sequential transitions among bactericidal, anti‐inflammatory, and antioxidative states, thereby aligning therapeutic actions with disease evolution.

**SCHEME 1:**
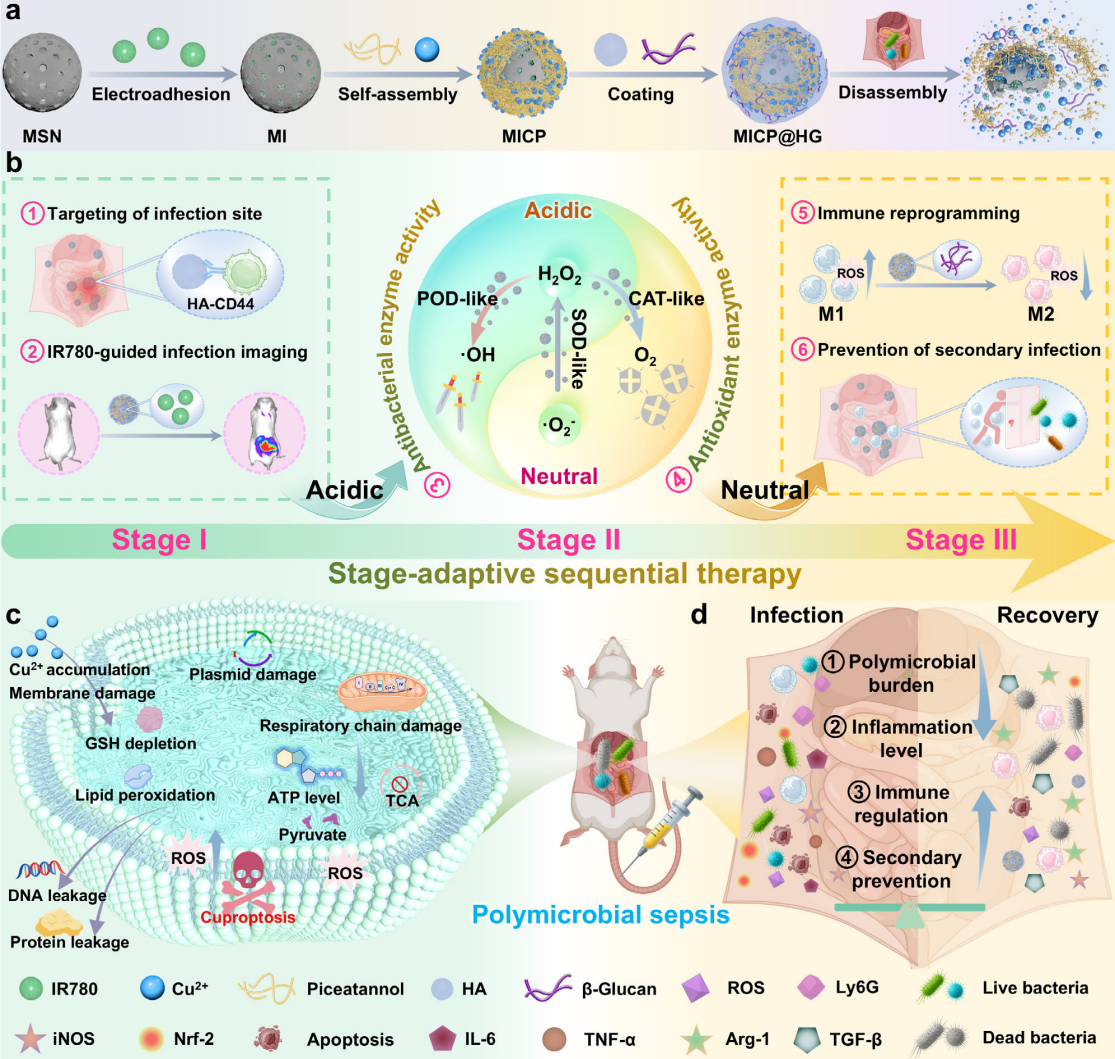
Schematic illustrations of the construction and application of MICP@HG. (a) Preparation of MICP@HG. (b) Multifunctional properties of MICP@HG. (c) Antibacterial mechanism of MICP@HG. (d) Anti‐inflammatory and immunomodulatory effects of MICP@HG in sepsis.

Here, we present a multifunctional nanoplatform that establishes a therapeutic framework that shifts sepsis intervention from static treatment toward stage‐adaptive therapy. The platform consists of a mesoporous silica (MSN) core loaded with the near‐infrared dye IR780 for real‐time infection imaging [20, 21, 22, 23, 24, 25, 26], a Cu‐piceatannol (CuP) metal‐polyphenol shell with pH‐responsive multienzyme‐like activities, and an outer hyaluronic acid/β‐glucan coating for infection‐site targeting and immune modulation. Under acidic conditions, the CuP shell exerts potent antibacterial activity through dual mechanisms of catalytic ROS amplification and cuproptosis‐like bacterial killing, thereby disrupting the tricarboxylic acid cycle and redox homeostasis [27, 28, 29, 30, 31, 32, 33, 34, 35, 36, 37, 38]. As the microenvironment shifts toward neutrality, the system transitions toward antioxidative and anti‐inflammatory activities, mitigating oxidative damage and facilitating immune restoration [39, 40, 41]. Meanwhile, hyaluronic acid (HA)‐mediated CD44 targeting and β‐glucan‐induced macrophage reprogramming further reinforce immune reprogramming and homeostasis. Therefore, the HA/β‐glucan coating was designed as a functional outer layer to combine infection‐site enrichment with immune regulation, rather than a new mechanism requiring independent redefinition in this study [42, 43, 44, 45, 46, 47, 48, 49, 50]. This study presents a microenvironment‐adaptive nanotherapeutic paradigm that synchronizes antibacterial, antioxidative, anti‐inflammatory, and immunoregulatory activities in a temporally controlled manner. By addressing the intrinsic heterogeneity and dynamic progression of sepsis, this strategy provides a promising framework for microenvironment‐guided precision therapy (Scheme 1).

## 2 Results

### 2.1 Synthesis and Characterization of MICP@HG

**FIGURE 1:**
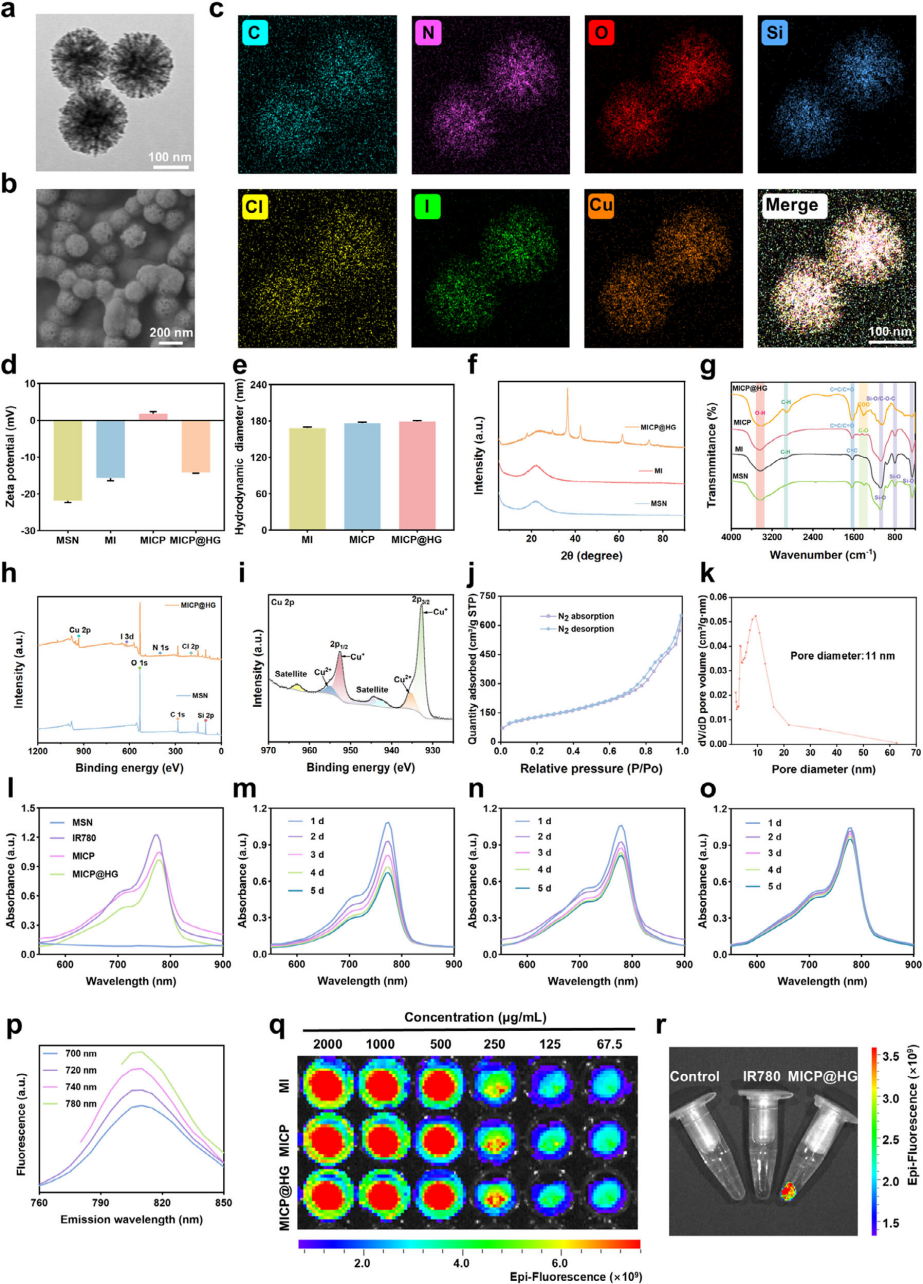
Material characterizations and spectral features of MICP@HG. (a) TEM image of MICP@HG. (b) SEM image of MICP@HG. (c) Element mappings of MICP@HG. (d) Zeta potentials of MSN, MI, MICP, and MICP@HG. (e) Dynamic light scattering of MI, MICP, and MICP@HG. (f) XRD patterns of MSN, MI, and MICP@HG. (g) FT‐IR spectra of MSN, MI, MICP, and MICP@HG. (h) XPS spectra of MSN and MICP@HG. (i) Cu 2p XPS spectra of MICP@HG. (j) Nitrogen adsorption–desorption curves of MSN. (k) Pore diameter distributions of MSN. (l) UV–vis–NIR absorption spectra of MSN, IR780, MICP, and MICP@HG. Optical stabilities of (m) IR780, (n) MICP, and (o) MICP@HG during a 5‐day storage period. (p) Fluorescence emission spectra of MICP@HG under varying excitation wavelengths. (q) Fluorescence images of MI, MICP, and MICP@HG at different concentrations under excitation. (r) Fluorescence images of control, IR780, and MICP@HG after excitation.

We first engineered a hierarchically integrated nanozyme platform (MICP@HG) for imaging‐guided and microenvironment‐responsive catalytic therapy. MSN@IR780 (MI) was prepared through electrostatic loading. MSN@IR780@CuP (MICP) was subsequently assembled via a one‐pot coordination process and further modified with hyaluronic acid (HA) and β‐glucan to obtain MICP@HG. TEM imaging revealed a well‐defined mesoporous architecture in MSN; the mesochannels became substantially obscured after hierarchical assembly in MICP@HG (Figure 1a; Figure S1a,b). The morphology of MSN and MICP@HG was examined by scanning electron microscopy (SEM), revealing that both samples exhibited a uniform spherical morphology. After sequential modification with CuP, HA, and β‐glucan, the nanoparticles maintained their spherical shape, while a distinct polymer coating layer became visible on the surface (Figure 1b; Figure S1c,d). The high‐angle annular dark‐field scanning TEM (HAADF‐STEM) image showed MICP@HG as bright white spots, and EDS elemental mapping confirmed the uniform distribution of Cu, C, N, O, Si, Cl, and I (Figure 1c; Figure S1e). The zeta potential analysis revealed a progressive surface charge reversal during synthesis. After coordination with CuP, the zeta potential of MSN reversed from negative to positive, confirming the incorporation of CuP. Subsequently, the potential shifted back to negative upon HA and β‐glucan modification, indicating that these negatively charged biomacromolecules were anchored onto the nanoparticle surface (Figure 1d). The hydrodynamic diameters of MICP and MICP@HG were approximately 176.4 and 179.3 nm, respectively, as determined by dynamic light scattering (DLS). In contrast, bare MSN exhibited a smaller average size (∼168.5 nm), indicating that the stepwise coordination and polymer modification slightly increased the particle size due to surface coating (Figure 1e). Broad diffuse peaks were observed for MSN and MI, confirming their amorphous nature, whereas MICP@HG exhibited emerging diffraction peaks, suggesting partial structural ordering following CuP coordination (Figure 1f). The FTIR spectra further verified the sequential assembly process of MICP@HG. The characteristic Si–O–Si stretching vibrations at ∼1086, 804, and 468 cm^−^^1^ confirmed the silica framework. Upon IR780 and CuP loading, new peaks at ∼1634 and ∼1454 cm^−^^1^ appeared, corresponding to the aromatic C═C and C─O stretching vibrations of polyphenol structures. After coating with HA and β‐glucan, enhanced peaks appeared at ∼3435 cm^−^^1^ (O─H), ∼2924 cm^−^^1^ (C─H), ∼1403 cm^−^^1^ (COO^−^ symmetric stretching from HA), and ∼1072 cm^−^^1^ (C─O─C stretching from polysaccharide backbones), confirming successful deposition of the HA/β‐glucan outer layer (Figure 1g). X‐ray photoelectron spectroscopy (XPS) analysis confirmed the presence of the elements Cu, C, N, O, Si, Cl, and I in MICP@HG (Figure 1h). From high‐resolution Cu 2p spectra, the peaks at 934.9/954.8 and 932.5/952.4 eV were assigned to Cu^2+^ and Cu^+^ (mixed‐valence copper), respectively (Figure 1i). Nitrogen adsorption–desorption analysis revealed that MSN possessed a well‐ordered mesoporous structure with an average pore diameter of 11 nm and a high Brunauer–Emmett–Teller (BET) surface area of 459 m^2^/g. Combined with its hollow cavity, these features endowed MSN with excellent loading capacity for IR780, CuP, HA, and β‐glucan, supporting its utility as a high‐capacity therapeutic carrier (Figure 1j,k). The spectral characteristics and optical stability of MICP@HG were further evaluated. UV–vis–NIR absorption spectra of MSN, IR780, MICP, and MICP@HG were recorded. Both MICP and MICP@HG displayed the characteristic absorption peaks of IR780 (Figure 1l). During a 5‐day storage period, the absorption intensity of free IR780 gradually declined due to its strong hydrophobicity and fluorescence self‐quenching, whereas MICP and MICP@HG maintained stable absorption. These results indicated that IR780 loading within the MSN framework enhanced its colloidal stability while suppressing self‐quenching. In addition, the coating of CuP, HA, and β‐glucan did not alter the intrinsic absorption characteristics of IR780 (Figure 1m–o). Notably, MICP@HG exhibited negligible spectral changes during storage, highlighting its potential as a robust theranostic nanoplatform. Fluorescence emission analysis revealed that the fluorescence intensity increased with increasing excitation wavelength (Figure 1p). The fluorescence intensity remained largely unchanged among MI, MICP, and MICP@HG, indicating that the surface functionalization did not affect the optical properties of IR780 (Figure 1q). Compared with free IR780 dispersed in PBS, MICP@HG exhibited enhanced fluorescence intensity, highlighting its improved optical stability and suitability for in vivo fluorescence imaging (Figure 1r). Free IR780 precipitated at the bottom of the tube because of its poor aqueous solubility. To allow comparison within the same imaging region, MICP@HG was also collected at the bottom by centrifugation before imaging; therefore, the localized fluorescence signal does not indicate poor dispersion of MICP@HG.

### 2.2 pH‐Responsive Enzyme‐Mimicking Activities of MICP@HG

**FIGURE 2:**
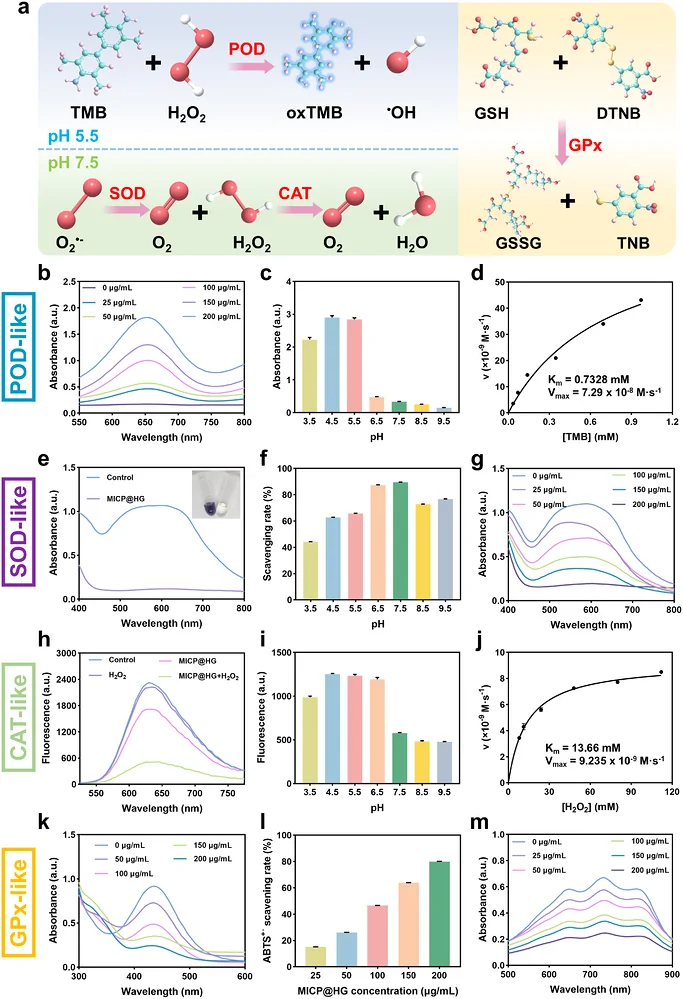
pH response of the glutathione peroxidase (GPx)‐, SOD‐, POD‐, and CAT‐like activities of MICP@HG. (a) Schematic diagram of multiple‐enzyme activities of MICP@HG. (b) Absorbance of oxidized TMB with different concentrations of MICP@HG. (c) Absorbance of oxidized TMB catalyzed by MICP@HG under different pH conditions. (d) Michaelis‐Menten curves of ROS generation velocities versus the concentration of TMB. (e) UV–vis absorption spectra of NBT and riboflavin in the presence of MICP@HG. (f) Catalytic conversion of •O_2_^−^ for the MICP@HG nanozyme at different pH. (g) UV–vis absorption spectra of NBT and riboflavin in the presence of different concentrations of MICP@HG. (h) Fluorescence spectra of oxygen generation under different treatment conditions. (i) Fluorescence intensity of oxygen generation under different pH conditions. (j) CAT‐like Michaelis‐Menten kinetic analysis of MICP@HG. (k) Concentration‐dependent GSH consumption of MICP@HG. (l) ABTS^+•^ scavenging rates of MICu@HG at different concentrations. (m) UV–vis absorption spectra of ABTS^+•^ after incubation with different concentrations of MICu@HG. Data are presented as the mean ± SD, n = 3. Group differences were analyzed by one‐way ANOVA followed by Tukey's HSD post‐hoc test. Significance is denoted as ^*^ *p* < 0.05, ^**^ *p* < 0.01, ^***^ *p* < 0.001, and ns indicates no significant difference (*p* ≥ 0.05).

Septic infectious microenvironments are characterized by profound redox dysregulation accompanied by dynamic pH fluctuations. Accordingly, dynamic regulation of ROS amplification and clearance at different stages of tissue repair is critical. The ROS‐producing and anti‐inflammatory capabilities of the MICP@HG nanozyme under various pH conditions were subsequently evaluated based on its multienzyme‐like catalytic activities (Figure 2a). Peroxidase (POD)‐mimicking nanozymes catalyze H_2_O_2_ decomposition to generate highly reactive oxidative species through Fenton‐like catalysis [51, 52]. A pronounced colorimetric change was observed when MICP@HG and H_2_O_2_ were added to an acidic solution containing TMB (Figure S2a). The absorbance increased significantly in a concentration‐dependent manner with MICP@HG (Figure 2b). To further evaluate the influence of pH, the POD‐like activity of MICP@HG was examined under alkaline conditions, where negligible activity was observed (Figure 2c). According to Michaelis–Menten kinetics, ROS generation increased with increasing TMB concentration (Figure 2d). The Michaelis–Menten constant (K_m_) and maximum reaction velocity (V_max_) of MICP@HG toward TMB were 0.7328 mM and 7.29 × 10^−^^8^ M s^−^^1^, respectively, based on Lineweaver–Burk plots (Figure S2b). When H_2_O_2_ was used as the substrate, the corresponding K_m_ and V_max_ values were 14.83 mM and 1.175 × 10^−^^7^ M s^−^^1^, respectively (Figure S2c,d). The nitroblue tetrazolium (NBT) assay was employed to evaluate the ability of MICP@HG to scavenge superoxide radicals (O_2_^•−^). In this reaction, O_2_^•−^ reduces NBT to formazan, which exhibits a broad absorption band from 550 to 700 nm (Figure 2e). Given the pH sensitivity of enzyme‐mimicking catalysis, we further investigated the effect of pH on the superoxides dismutase (SOD)‐like activity of MICP@HG. MICP@HG exhibited the highest SOD‐like activity under mildly acidic and near‐neutral conditions (Figure 2f). The absorbance gradually decreased with increasing concentrations of MICP@HG (Figure 2g), confirming efficient superoxide scavenging capability.

Catalase (CAT)‐mimicking activity is critical for decomposing excessive H_2_O_2_, thereby eliminating excessive oxidants generated during the reduction of superoxide radicals [53]. Oxygen generation was monitored using [Ru(dpp)_3_]Cl_2_ to evaluate the CAT‐like activity of MICP@HG. The fluorescence of the probe decreased progressively with increasing MICP@HG concentration, indicating enhanced oxygen generation (Figure 2h; Figure S3a). To further investigate the influence of pH, the CAT‐like activity of MICP@HG was examined under different pH conditions. MICP@HG exhibited pronounced CAT‐mimicking activity under near‐neutral and mildly alkaline conditions, whereas its activity was markedly suppressed under acidic conditions, indicating pH‐responsive catalytic behavior (Figure 2i). The clearance reaction between MICP@HG and H_2_O_2_ followed Michaelis‐Menten kinetics (Figure 2j; Figure S3b), with a V_max_ of 9.235 × 10^−9^ M s^−1^. Glutathione (GSH) serves as a major intracellular redox buffer that maintains intracellular redox homeostasis in bacterial cells. The GSH consumption capability of MICP@HG was evaluated using 5,5′‐dithiobis(2‐nitrobenzoic acid) (DTNB). After MICP@HG was co‐incubated with GSH, the intensity of the characteristic peaks attributed to the combination of GSH and DTNB gradually decreased and eventually disappeared (Figure S3c). The characteristic peak at 415 nm exhibited a concentration‐dependent decrease in the presence of MICP@HG (Figure 2k). To further validate the radical‐scavenging capability of MICP@HG, a free radical scavenging assay was conducted using the 2,2′‐azino‐bis(3‐ethylbenzothiazoline‐6‐sulfonic acid) (ABTS•^+^) method. The characteristic green‐blue ABTS•^+^ undergoes decolorization upon interaction with antioxidants, reflecting their radical scavenging efficiency. As the concentration of MICP@HG increased, the ABTS•^+^ scavenging rate increased correspondingly, indicating a dose‐dependent antioxidant effect. More than 80% of the free radicals were eliminated even at a relatively low concentration of 200 µg/mL, demonstrating the potent free‐radical scavenging activity of MICP@HG (Figure 2l,m).

To verify whether MICP@HG dynamically adapts its catalytic functions during disease progression, we established an in vitro microenvironment model that mimics the transition from the acidic infection stage to the near‐neutral inflammatory resolution stage (Figure S3d). Briefly, bacteria were incubated with MICP@HG in an acidic medium for the first 2 h to simulate the early infectious microenvironment. The medium was subsequently adjusted to physiological pH while RAW 264.7 macrophages cultured in the lower chamber were continuously exposed to MICP@HG, thereby recapitulating the transition from bacterial infection to the resolution of inflammation. During the acidic infection stage, intracellular ROS levels in bacteria increased continuously over time, indicating sustained pro‐oxidative antibacterial activity of MICP@HG (Figure S3e). Following pH adjustment to a neutral environment, intracellular ROS levels in RAW 264.7 macrophages gradually decreased (Figure S3f), demonstrating a shift from ROS generation toward ROS scavenging. These results demonstrate that MICP@HG dynamically reprograms its catalytic behavior according to disease‐associated microenvironmental evolution, enabling oxidative antibacterial activity during early infection while exerting antioxidant and anti‐inflammatory effects during the recovery phase.

### 2.3 Antibacterial Activity Test In Vitro

**FIGURE 3:**
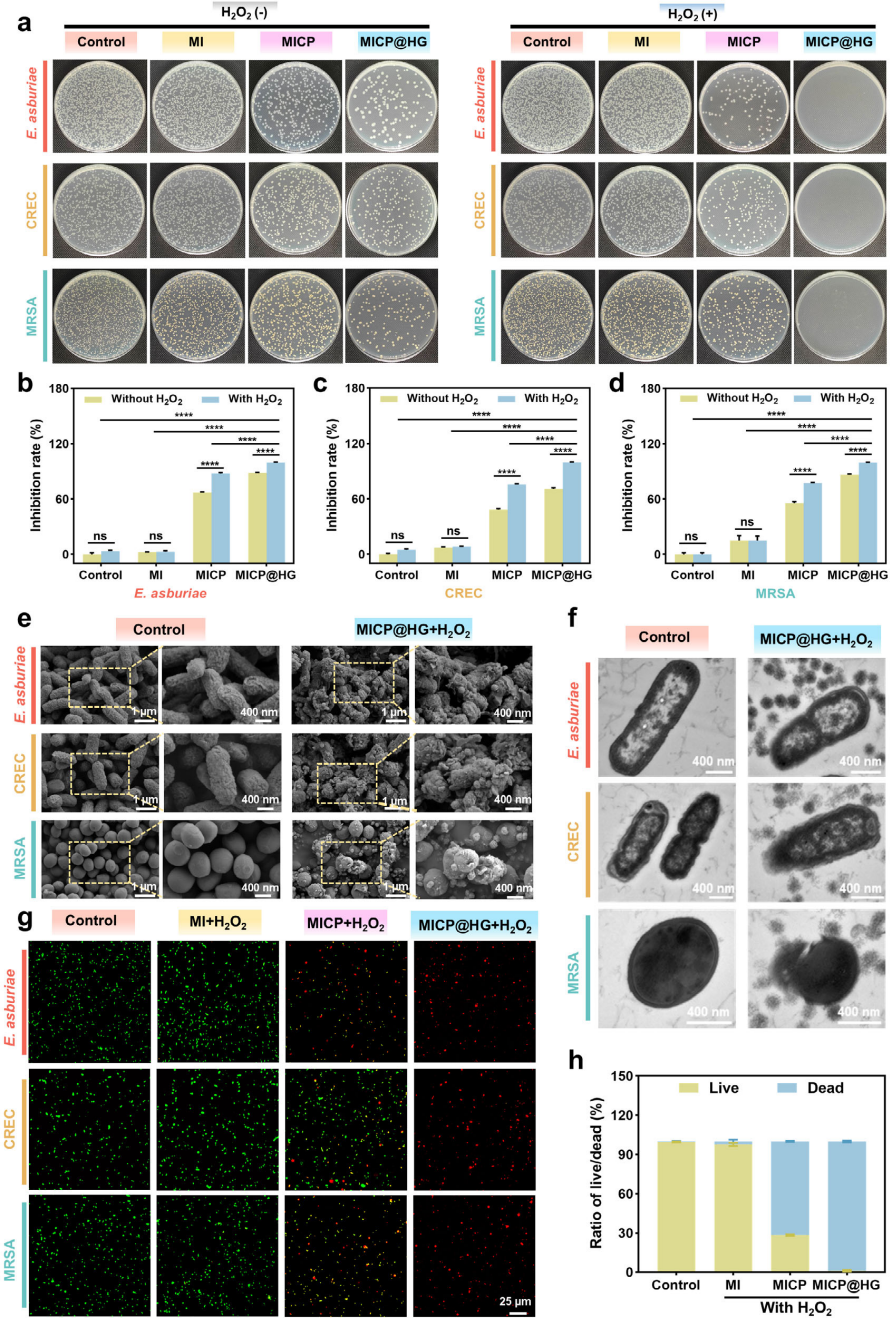
Antibacterial activity against *E. asburiae*, CREC, and MRSA in vitro. (a–d) Representative agar plate photographs and quantitative analysis of *E. asburiae*, CREC, and MRSA after different treatments. (e) SEM morphologies of *E. asburiae*, CREC, MRSA after different treatments. (f) TEM morphologies of *E. asburiae*, CREC, MRSA after different treatments. (g) Representative fluorescence photos of *E. asburiae*, CREC, MRSA stained with calcein‐AM (green, live bacteria) and PI (red, dead bacteria) in different groups. (h) Quantitative analysis of *E. asburiae* stained with calcein‐AM (green, live bacteria) and PI (red, dead bacteria) in different groups. Data are presented as mean ± SD, n = 3. Group differences were analyzed by one‐way ANOVA followed by Tukey's HSD post‐hoc test. Significance is denoted as ^*^ *p* < 0.05, ^**^ *p* < 0.01, ^***^ *p* < 0.001, and ns indicates no significant difference ( ≥ 0.05).

Given the polymicrobial nature of sepsis‐associated infections, the broad‐spectrum antibacterial activity of MICP@HG was comprehensively investigated. The antimicrobial efficacy was tested against clinically relevant pathogens, including Gram‐positive methicillin‐resistant *Staphylococcus aureus* (MRSA), Gram‐negative carbapenem‐resistant *Escherichia coli* (CREC), and the super multidrug‐resistant strain *E. asburiae*. The antibacterial performance of PBS, MI, MICP, and MICP@HG was examined using the agar plate colony assay (Figure 3a). Notably, MICP@HG combined with H_2_O_2_ exhibited potent broad‐spectrum bactericidal activity against all tested bacteria, whereas the control and MI groups (with or without H_2_O_2_) exhibited minimal antibacterial effects. Furthermore, MICP@HG displayed significantly enhanced antibacterial activity compared with MICP, both in the presence and absence of H_2_O_2_, which likely resulted from enhanced bacteria–nanozyme interfacial interactions mediated by HA functionalization (Figure 3b–d). This HA‐mediated targeting facilitated intimate interfacial contact between the nanocomposite and bacterial membranes, thereby enhancing localized catalytic ROS generation and overall antibacterial performance. Both the bacterial colony‐count assay and inhibition‐rate analysis demonstrated that the MICP@HG/H_2_O_2_ treatment displayed substantially enhanced bactericidal efficacy compared with MICP@HG alone. This enhancement was attributed to H_2_O_2_‐triggered activation of POD‐like catalytic pathways, thereby amplifying ROS generation responsible for bacterial killing.

The antibacterial effect of MICP@HG was further investigated using SEM and TEM to visualize bacterial morphology after different treatments. MRSA, CREC, and *E. asburiae* cells exposed to MICP@HG/H_2_O_2_ exhibited severe structural damage, including membrane shrinkage, collapse, and fusion, indicating catastrophic membrane and cell wall damage (Figure 3e,f). Notably, we observed abundant nanocomposite adhesion on the bacterial surfaces following MICP@HG/H_2_O_2_ exposure, suggesting that HA‐mediated targeting enhanced contact‐mediated catalytic bactericidal activity. In addition, live/dead staining with PI and calcein‐AM (Figure 3g; Figure S4a) revealed that bacterial viability was lowest in the MICP@HG/H_2_O_2_ group, moderate in the MICP@HG group, and highest in the H_2_O_2_ and control groups, consistent with the quantitative antibacterial results (Figure 3h; Figure S4b,c). These findings collectively confirmed that MICP@HG exerted potent antibacterial activity through H_2_O_2_‐activated catalytic ROS generation and membrane disruption.

### 2.4 Cuproptosis‐Like Bacterial Death In Vitro

**FIGURE 4:**
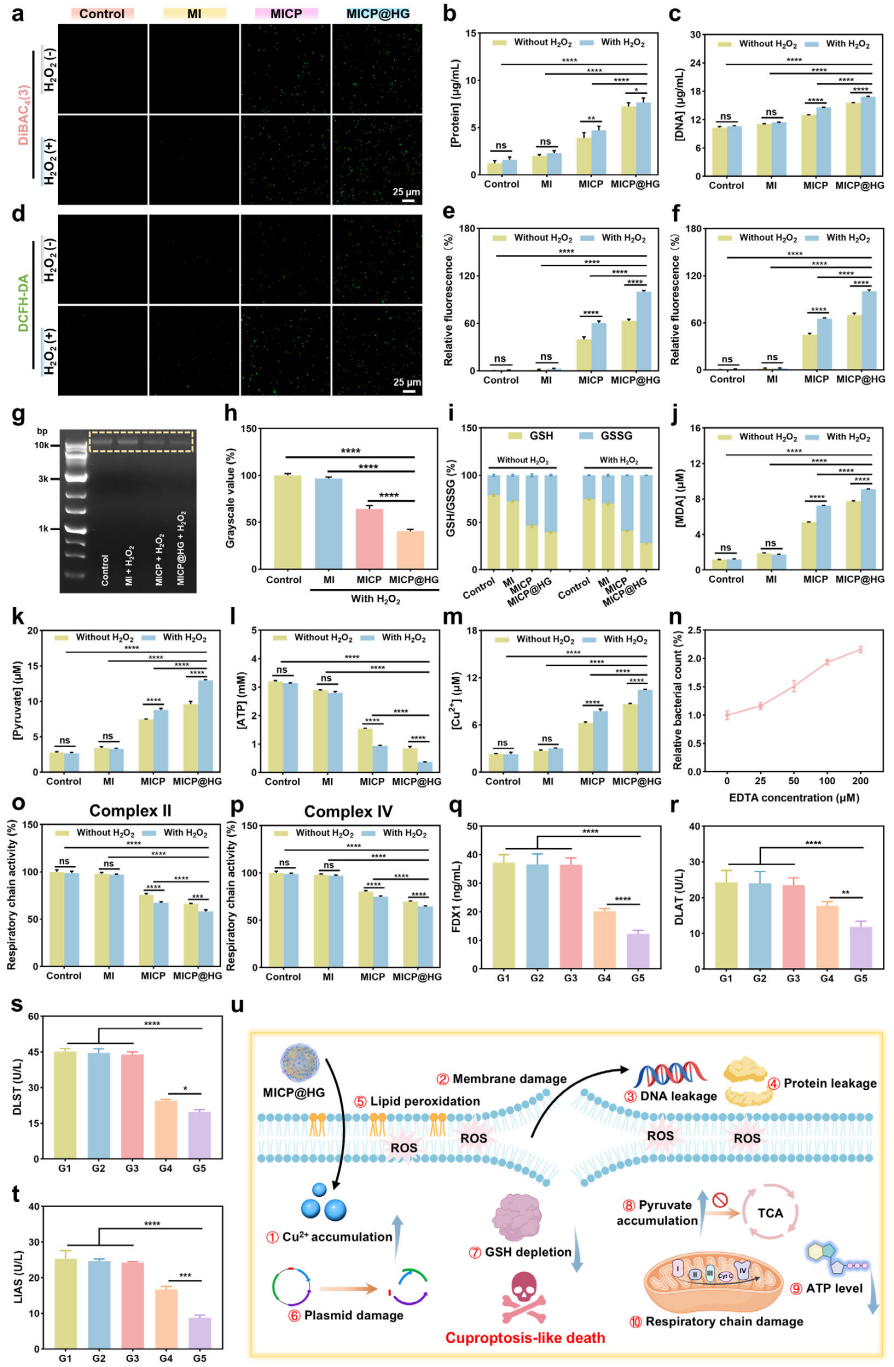
Cuproptosis‐like death of *E. asburiae* in vitro. (a,e) Representative DiBAC_4_(3) staining images and quantitative analysis of *E. asburiae* after different treatments. (b) Extracellular protein concentrations of *E. asburiae* after different treatments. (c) Extracellular nucleic acid concentrations of *E. asburiae* after different treatments. (d, f) Representative staining fluorescence photographs and quantitative analysis of ROS (green) in *E. asburiae* detected by DCFH‐DA after different treatments. (g) Agarose gel electrophoresis of plasmid extracted from *E. asburiae* after different treatments and (h) corresponding quantitative analysis. (i) Ratios of GSH/GSSG, (j) lipid peroxidation levels, (k) pyruvate contents, (l) intracellular ATP levels in *E. asburiae* after different treatments. (m) Copper concentration statistics of *E. asburiae* after different treatments. (n) Effect of a cuproptosis death inhibitor (EDTA) on the anti‐*E. asburiae* activity of MICP@HG. (o, p) Influence of different treatments on the activity of *E. asburiae* respiratory chain complexes II and IV. Expression levels of cuproptosis‐related markers including (q) FDX1, (r) DLAT, (s) DLST, and (t) LIAS following treatment. (G1: Control; G2: H_2_O_2_; G3: MICP@HG + H_2_O_2_ + EDTA; G4: MICP@HG + H_2_O_2_ + VC; G5: MICP@HG + H_2_O_2_). (u) Schematic diagram of the antibacterial mechanism of MICP@HG. Data are presented as mean ± SD, n = 3. Group differences were analyzed by one‐way ANOVA followed by Tukey's HSD post‐hoc test. Significance is denoted as ^*^ *p* < 0.05, ^**^ *p* < 0.01, ^***^ *p* < 0.001, and ns indicates no significant difference (*p* ≥ 0.05).

To further investigate the copper‐dependent bactericidal mechanism in *E. asburiae*, MRSA, and CREC, we performed additional antibacterial mechanism analyses. Using the voltage‐sensitive fluorescent dye bis‐(1,3‐dibutylbarbituric acid)‐trimethine oxonol (DiBAC_4_(3)), membrane depolarization was monitored via green fluorescence, which arises upon dye entry into depolarized cells and binding to intracellular components. We observed strong green fluorescence exclusively in the MICP@HG/H_2_O_2_‐treated bacteria, indicating severe membrane depolarization (Figure 4a,e; Figures S5a,e and S6a,e). Such membrane depolarization was accompanied by increased membrane permeability and severe disruption of cellular integrity. Subsequently, extracellular proteins and nucleic acids were quantified to evaluate membrane damage. Consistent with the fluorescence results, the MICP@HG/H_2_O_2_ group showed the highest levels of extracellular proteins and nucleic acids, indicating substantial cytoplasmic leakage (Figure 4b,c; Figures S5b,c and S6b,c). These findings demonstrated that MICP@HG/H_2_O_2_ induced cuproptosis‐like bacterial death through membrane depolarization, structural disruption, and intracellular content leakage.

Bacterial redox homeostasis is maintained through the balance between ROS production and antioxidant defense mechanisms [54]. To evaluate oxidative stress, we monitored intracellular ROS levels in *E. asburiae*, MRSA, and CREC using the fluorescent probe DCFH‐DA. The PBS control, H_2_O_2_, and MI groups showed negligible fluorescence, indicating minimal ROS generation. Similarly, both MICP and MICP@HG groups exhibited low fluorescence signals under H_2_O_2_‐free conditions, suggesting limited oxidative activity. In contrast, the addition of H_2_O_2_ to the MICP group resulted in a moderate increase in intracellular ROS levels, reflecting its intrinsic POD‐like catalytic activity and partial antibacterial efficacy. Notably, the MICP@HG/H_2_O_2_‐treated bacteria displayed the strongest green fluorescence, corresponding to the highest intracellular ROS accumulation (Figure 4d,f; Figures S5d,f and S6d,f). MICP@HG effectively catalyzed H_2_O_2_ decomposition to generate highly reactive radicals, thereby inducing profound oxidative stress‐mediated bacterial injury.

To elucidate the impact of ROS on bacterial plasmids, we focused on *E. asburiae*, a super multidrug‐resistant strain harboring two major resistance genes on plasmids. We examined plasmid integrity following different treatments using agarose gel electrophoresis. Extensive plasmid degradation was observed after MICP@HG/H_2_O_2_ treatment, whereas intact plasmid bands remained visible in the control and MI/H_2_O_2_ groups (Figure 4g). Only trace amounts of intact plasmid DNA were detected in the MICP@HG/H_2_O_2_ group, indicating that ROS generated during catalytic reactions induced severe plasmid cleavage and structural damage. These results demonstrated that the strong oxidative stress induced by MICP@HG/H_2_O_2_ led to bacterial DNA fragmentation, potentially contributing to resistance gene elimination and efficient bacterial eradication (Figure 4h).

Given the GPx‐like activity of MICP@HG for GSH depletion, we measured the GSH/GSSG ratio to assess redox homeostasis. Both MICP@HG and MICP@HG/H_2_O_2_ treatments significantly disrupted redox balance, indicating impaired intracellular redox buffering (Figure 4i; Figures S5g and S6g). The level of membrane lipid peroxidation in bacteria was further evaluated using the malondialdehyde (MDA) assay. MDA levels increased in the MICP, MICP@HG, MICP/H_2_O_2_, and MICP@HG/H_2_O_2_ groups, with the MICP@HG/H_2_O_2_ group exhibiting the highest level, indicating severe lipid peroxidation and membrane damage (Figure 4j; Figures S5h and S6h).

Regarding bacterial energy metabolism, pyruvate detection revealed significant accumulation of pyruvate in the MICP@HG/H_2_O_2_‐treated group (Figure 4k; Figures S5i and S6i), whereas intracellular ATP content was markedly reduced (Figure 4l; Figures S5j and S6j). These findings indicated a profound metabolic dysfunction, consistent with disruption of tricarboxylic acid (TCA) cycle activity. Subsequent copper ion quantification further confirmed substantial intracellular copper accumulation in the MICP@HG/H_2_O_2_ group (Figure 4m; Figures S5k and S6k). Ethylenediaminetetraacetic acid (EDTA), a well‐known divalent metal‐ion chelator, forms complexes with Cu^2^^+^ and inhibits cuproptosis‐like death. We therefore used EDTA to verify whether copper‐dependent cuproptosis‐like processes contributed to bacterial killing. Notably, EDTA attenuated the bactericidal activity of MICP@HG in a concentration‐dependent manner (Figure 4n; Figures S5l and S6l), supporting the involvement of copper‐dependent cytotoxicity. Consistent with metabolic disruption, MICP@HG/H_2_O_2_ suppressed the activity of respiratory chain complexes II and IV (Figure 4o,p; Figures S5m,n and S6m,n), suggesting profound respiratory chain dysfunction and impaired oxidative phosphorylation.

To delineate the respective contributions of oxidative damage and cuproptosis‐like death to the bactericidal activity, ascorbic acid (VC) and EDTA were used as an antioxidant and a copper‐chelating inhibitor, respectively. VC scavenges ROS, including superoxide anions and hydroxyl radicals, thereby alleviating oxidative stress. By contrast, EDTA sequesters Cu^2+^ through stable complex formation, limiting its cellular uptake and subsequent interaction with lipoylated proteins [55]. Antibacterial assays showed that H_2_O_2_ alone exerted negligible bactericidal activity, whereas MICP@HG in the presence of H_2_O_2_ achieved a 100% antibacterial rate. VC reduced the antibacterial rate to approximately 68%, indicating that cuproptosis‐like death contributes substantially to MICP@HG‐mediated bacterial killing. Notably, EDTA showed a more pronounced protective effect, decreasing the antibacterial rate to approximately 35% (Figure S7). These results indicated that oxidative stress and copper‐dependent cuproptosis‐like death act synergistically to mediate bacterial killing, with oxidative damage further potentiating the copper‐dependent death pathway. To further substantiate this mechanism, FDX1, DLAT, DLST and LIAS were selected as representative markers of cuproptosis‐like death. ELISA showed that treatment with MICP@HG + VC significantly decreased the expression of all markers. In the MICP@HG + EDTA group, however, their expression levels were comparable to those in the control group, indicating that the downregulation resulted from cuproptosis‐like death (Figure 4q–t). The oxidative damage disrupted copper‐transport‐associated proteins, promoting aberrant intracellular Cu^2+^ accumulation, which in turn potentiated cuproptosis‐like death and further aggravated bacterial killing. In summary, MICP@HG/H_2_O_2_ induced cuproptosis‐like bacterial death, characterized by excessive ROS generation, disruption of GSH homeostasis, enhanced lipid peroxidation, copper accumulation, and suppression of the respiratory chain (Figure 4u). These events synergistically trigger redox imbalance and metabolic collapse, ultimately leading to irreversible bacterial destruction.

### 2.5 Transcriptomic Analysis of *E. asburiae* Following MICP@HG/H_2_O_2_ Treatment

**FIGURE 5:**
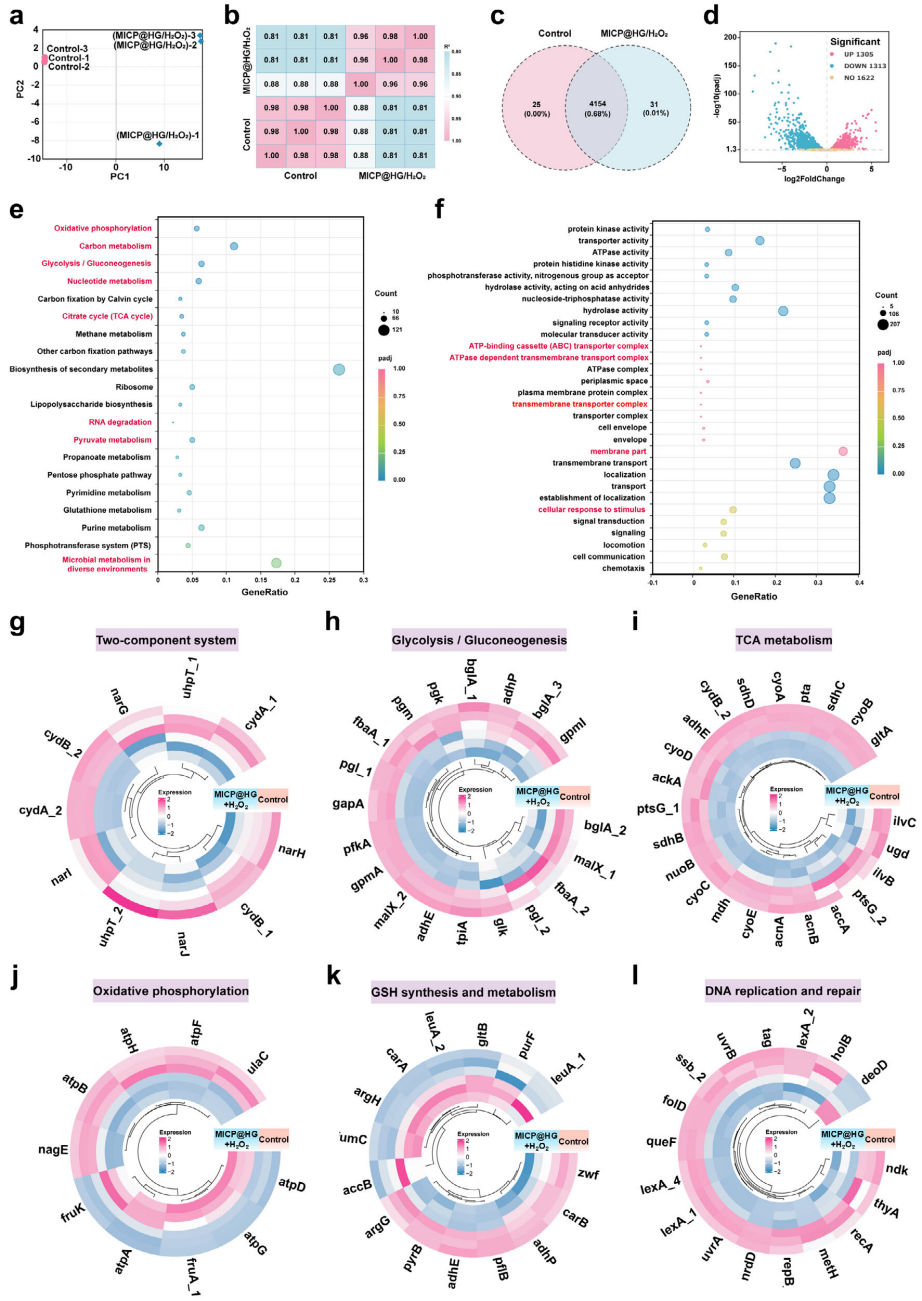
Regulation of the *E. asburiae* transcriptome by MICP@HG. (a) PCA analysis of the control and MICP@HG/H_2_O_2_ groups. (b) Correlation analysis of control groups and MICP@HG/H_2_O_2_ groups. (c) Venn diagram of DEGs between the control and MICP@HG/H_2_O_2_ groups. (d) Volcano plot analyses of total DEGs in *E. asburiae* after treatment withthe control or MICP@HG/H_2_O_2_. (e) KEGG enrichment analysis of DEGs. (f) GO enrichment analysis of DEGs. (g) Heat map of genes associated with TCS. (h) Heat map of genes associated with glycolysis/gluconeogenesis. (i) Heat map of genes associated with TCA cycle and pyruvate metabolism. (j) Heat map of genes associated with oxidative phosphorylation. (k) Heat map of genes associated with GSH synthesis and metabolism. (l) Heat map of genes associated with DNA replication and repair.

To further elucidate the systems‐level antibacterial mechanism, mRNA‐seq analysis was conducted on *E. asburiae* treated with MICP@HG/H_2_O_2_ and the untreated control. Principal component analysis (PCA) revealed a separation between the two groups, indicating profound transcriptomic reprogramming (Figure 5a). Correlation analysis further confirmed the strong intergroup reproducibility of the sequencing data (Figure 5b). As shown in the volcano plot (Figure 5c), a total of 2618 differentially expressed genes (DEGs) were identified, including 1305 upregulated and 1313 downregulated genes in the MICP@HG/H_2_O_2_ group compared with the control (Figure 5d). Based on these DEGs, Kyoto Encyclopedia of Genes and Genomes (KEGG) enrichment analysis revealed that MICP@HG/H_2_O_2_ predominantly disrupted bacterial metabolic and bioenergetic pathways, including oxidative phosphorylation, carbon metabolism, glycolysis/gluconeogenesis, nucleotide metabolism, the tricarboxylic acid (TCA) cycle, RNA degradation, and pyruvate metabolism (Figure 5e). Gene Ontology (GO) enrichment analysis further showed that oxidoreductase‐related pathways exhibited the most pronounced transcriptional perturbations, while those involved in metal ion stress and membrane damage responses were also significantly enriched (Figure 5f). Collectively, MICP@HG/H_2_O_2_ induces global disruption of bacterial metabolic and redox homeostasis, particularly affecting the ATP‐binding cassette (ABC) transporter complex, ATP‐dependent transmembrane transport systems, and cellular stress responses.

To further elucidate the functional networks disrupted by MICP@HG/H_2_O_2_, heatmap analysis was conducted to visualize transcriptional alterations in key metabolic and regulatory systems. The bacterial two‐component system (TCS) is a vital signal transduction mechanism that enables bacteria to sense environmental changes, regulate adaptive responses, and control toxin expression for survival under stress [56, 57]. Heatmap analysis revealed significant downregulation of TCS‐related genes following MICP@HG/H_2_O_2_ treatment (Figure 5g), suggesting that the nanocatalytic stress impaired bacterial environmental sensing and stress adaptation capacity. Copper overload has been associated with disruption of the TCA cycle and promoting lipid peroxide accumulation, thereby triggering cuproptosis‐like death [34]. Consistently, genes involved in the TCA cycle, glycolysis/gluconeogenesis, and oxidative phosphorylation were broadly downregulated in the MICP@HG/H_2_O_2_ group (Figure 5h–j), indicating profound bioenergetic suppression. Moreover, genes responsible for glutathione (GSH) biosynthesis were downregulated, whereas those associated with GSH degradation and oxidative stress response were upregulated (Figure 5k). This transcriptional pattern indicates pronounced redox imbalance, further supporting that MICP@HG/H_2_O_2_ promotes copper‐associated oxidative metabolic collapse. During copper‐associated oxidative stress, excessive ROS are generated, imposing extensive oxidative burden on bacterial cells. The accumulated ROS oxidize membrane lipids, denature proteins, deactivate key metabolic enzymes, and damage nucleic acids, ultimately impairing essential cellular functions [58]. Consistently, transcriptomic analysis revealed a pronounced downregulation of genes involved in DNA replication and repair pathways in the MICP@HG/H_2_O_2_ group (Figure 5l), indicating ROS‐induced oxidative DNA damage and defective genomic maintenance and DNA repair capacity.

### 2.6 Antioxidant, Anti‐Inflammatory, and Immunomodulatory Activities of MICP@HG In Vitro

**FIGURE 6:**
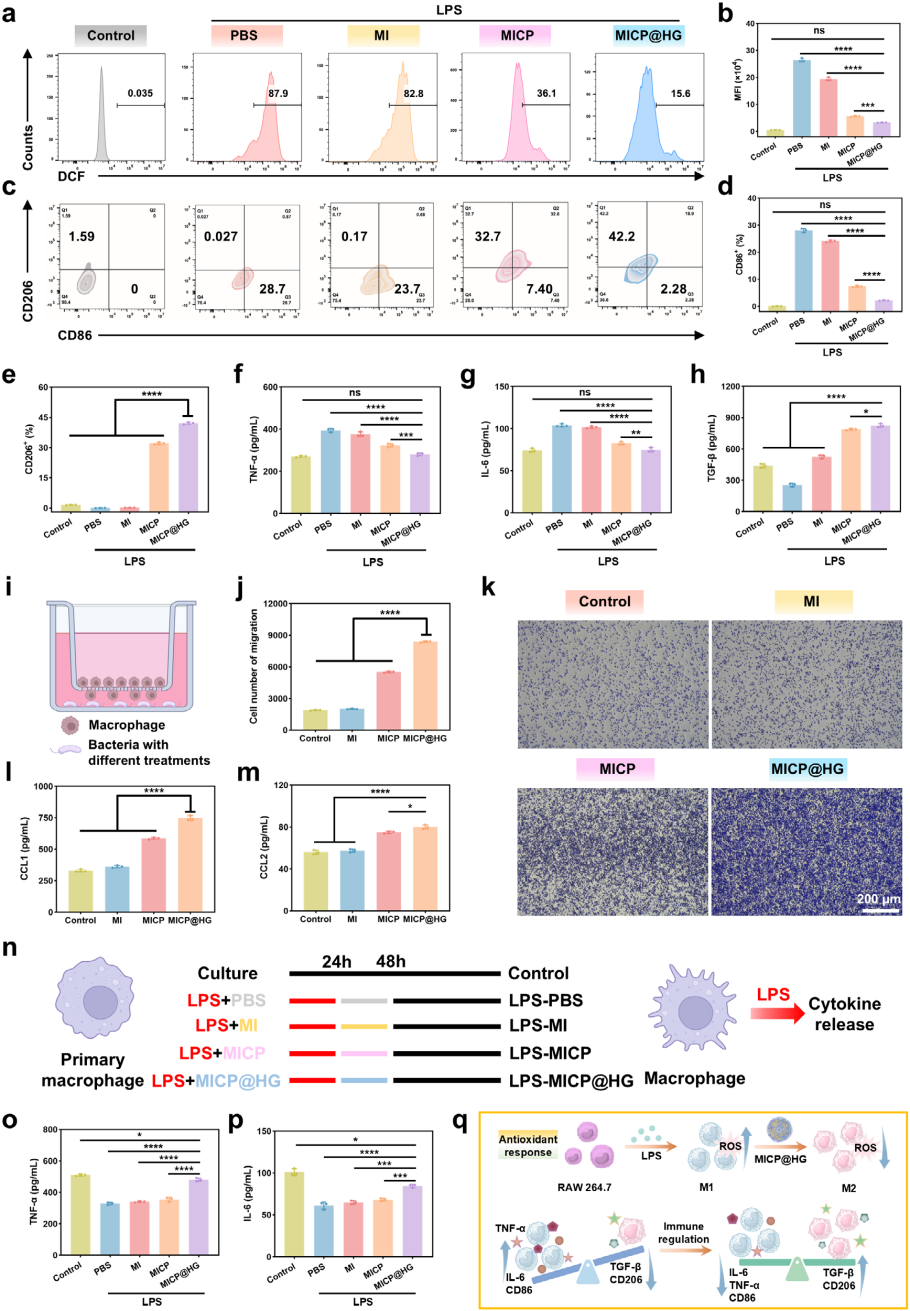
In vitro antioxidant activity and anti‐inflammatory effects. (a) Flow cytometry study of RAW 264.7 cells stained with DCFH‐DA. (b) Mean fluorescence intensity (MFI) of ROS levels in RAW 264.7 cells with different treatments. (c) Phenotype of RAW 264.7 cells determined by flow cytometry. (d) The mean percentages of M1‐type macrophages. (e) Mean percentages of M2‐type macrophages. Expression levels of (f) TNF‐α, (g) IL‐6, and (h) TGF‐β secreted by RAW 264.7 cells with different treatments. (i) Schematic diagram of cell chemotaxis transwell test. (j) Statistical analysis of transwell test data. (k) Transwell test cell staining. Expression levels of (l) CCL1 and (m) CCL2 secreted by RAW 264.7 cells with different treatments. (n) In vitro primary macrophage tolerance reversal model, with different treatments added therapeutically after 24 h of LPS exposure. Following several days of rest, macrophages were re‐exposed to LPS and cytokine release was measured after 24 h. (o) MICP@HG re‐instates TNF‐α release in tolerized macrophages. (p) MICP@HG re‐instates IL‐6 release in tolerized macrophages. (q) Schematic illustration of the antioxidant activity and anti‐inflammatory effect at the cellular level. Data are presented as mean ± SD, n = 3. Group differences were analyzed by one‐way ANOVA followed by Tukey's HSD post‐hoc test. Significance is denoted as ^*^ *p* < 0.05, ^**^ *p* < 0.01, ^***^ *p* < 0.001, and ns indicates no significant difference (*p* ≥ 0.05).

Excessive ROS accumulation is a hallmark of sepsis‐associated inflammation, leading to oxidative cellular injury and amplification of inflammatory signaling. To further evaluate the antioxidant potential of MICP@HG beyond its intrinsic SOD‐ and CAT‐like catalytic activities, we examined its ability to alleviate intracellular oxidative stress at the cellular level. An in vitro oxidative stress model was established by stimulating RAW 264.7 macrophages with lipopolysaccharide (LPS), followed by quantification of intracellular ROS using 2′,7′‐dichlorofluorescein diacetate (DCFH‐DA) and flow cytometry (Figure 6a,b). LPS stimulation markedly elevated intracellular ROS levels compared with the control group. Notably, treatment with MICP@HG significantly attenuated ROS accumulation, exhibiting superior antioxidant efficacy compared with other formulations. This pronounced reduction in ROS generation demonstrates that MICP@HG effectively alleviates oxidative stress, protects cells from oxidative damage, and mitigates inflammation‐associated injury.

To evaluate whether MICP@HG modulates macrophage polarization, flow cytometry analysis was conducted to examine the expression of phenotypic markers CD86 and CD206 on RAW 264.7 cells. LPS stimulation markedly upregulated CD86 expression, confirming the induction of pro‐inflammatory M1 macrophages. In contrast, cells treated with MICP@HG exhibited a significant increase in CD206 expression with a concomitant decrease in CD86 levels (Figure 6c–e), suggesting a phenotypic shift toward the anti‐inflammatory M2 subtype. To further verify this transition, the secretion of representative cytokines associated with M1 (TNF‐α, IL‐6) and M2 (TGF‐β) phenotypes was quantified by ELISA. Compared with the PBS, MI and MICP groups, MICP@HG treatment resulted in significantly elevated TGF‐β secretion and suppressed levels of TNF‐α and IL‐6 levels (Figure 6f–h). MICP@HG effectively reprogrammed macrophages from a pro‐inflammatory (M1) to an anti‐inflammatory (M2) phenotype, thereby facilitating inflammatory resolution and immune homeostasis and attenuating inflammatory signaling. MICP@HG establishes a redox–immune regulatory axis that bridges catalytic activity and host immune homeostasis, providing a promising strategy for synchronized antibacterial and immunoregulatory therapy. To evaluate the immunomodulatory recruitment capacity of MICP@HG in vitro, a transwell chemotaxis assay was performed. Specifically, bacteria subjected to different treatments were placed in the lower chamber to mimic an inflammatory microenvironment, while RAW 264.7 macrophages were seeded in the upper chamber to assess their chemotactic migration in response to different treatments (Figure 6i). Macrophages, as innate immune effector cells, play a pivotal role in pathogen clearance and immune orchestration by migrating toward infection sites and orchestrating antimicrobial immune responses [59]. Consistent with this physiological function, MICP@HG treatment markedly enhanced macrophage migration across the transwell membrane compared with other treatment groups (Figure 6j,k), indicating enhanced chemotactic recruitment capability. To further elucidate the underlying mechanism, the culture supernatants were collected after different treatments and analyzed for the secretion of chemotactic cytokines by ELISA. The levels of chemokines CCL1 and CCL2, which regulate macrophage migration and localization, were significantly upregulated upon MICP@HG treatment (Figure 6l,m). MICP@HG not only modulated macrophage polarization but also promoted coordinated immune cell recruitment to infection sites. This integrated immunoregulatory functionality enables coordinated spatial recruitment and functional immune regulation, thereby facilitating efficient pathogen clearance while limiting excessive inflammation.

Given that β‐glucan possesses the intrinsic ability to modulate macrophage immune tolerance, and that recurrent bacterial infections often trigger a state of macrophage desensitization, we further evaluated whether MICP@HG could restore innate immune responsiveness under tolerant conditions. Primary macrophages were first stimulated with LPS for 24 h to induce an immune‐tolerant phenotype, followed by treatment with MI, MICP, or MICP@HG for another 24 h. After a rest phase, the cells were re‐exposed to LPS, and cytokine secretion was quantified after 24 h (Figure 6n). As shown in Figure 6o,p, macrophages pretreated with MICP@HG exhibited a marked restoration of cytokine production compared with the tolerance groups. Specifically, MICP@HG exposure reversed LPS‐induced suppression of cytokine secretion, substantially rescuing cytokine responsiveness, whereas MICP treatment elicited only partial recovery. MICP@HG effectively reprogrammed macrophages from an immune‐tolerant to a responsive state. Importantly, MICP@HG not only alleviated oxidative stress and modulated polarization but also restored innate immune responsiveness, highlighting its potential as a therapeutic strategy for overcoming post‐infection immune tolerance and preventing infection‐associated immune paralysis and recurrent infection (Figure 6q). The cytotoxicity and biocompatibility of MICP@HG were evaluated in HUVECs and RAW 264.7 macrophages. Both the pristine materials and MICP@HG exhibited negligible cytotoxicity after 24 and 48 h of incubation. Cell viability remained about 100% across all tested concentrations, confirming the excellent biocompatibility of MICP@HG for subsequent infection‐related therapeutic applications (Figure S8).

### 2.7 In Vivo Targeting and Therapeutic Efficacy of MICP@HG in Polymicrobial Sepsis

**FIGURE 7:**
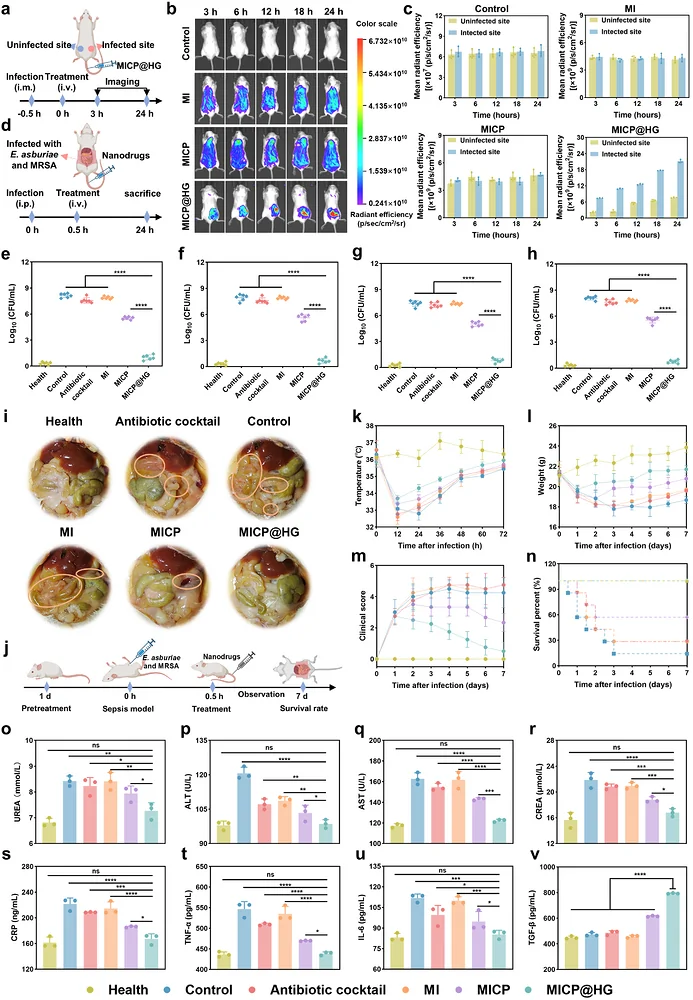
In vivo targeting and therapeutic efficacy of MICP@HG in polymicrobial sepsis models. (a) Schematics of the process to evaluate the targeting function of MICP@HG in a mouse muscle infection model. (b) Representative fluorescence images of the infected mice under different treatments at 3, 6, 12, 18, and 24 h after injection. (c) Mean fluorescence intensity of the infected and the uninfected sites in different treatment groups (n = 3). (d) Schematics of the process to evaluate the therapeutic efficacy of MICP@HG (100 µg/mL) in treating mice with *E. asburiae* and MRSA‐induced sepsis. Quantification of the number of bacteria in (e) peritoneal fluid, (f) liver, (g) lung, and (h) kidney of the infected mice with different treatments. Infected mice treated with PBS were used as controls (n = 6). (i) Abdominal view of septic mice with different treatments. (j) Schematic illustration of the experimental design to evaluate the therapeutic efficacy of MICP@HG (100 µg/mL) in mice with *E. asburiae* and MRSA‐induced sepsis over a 7‐day observation period. (k) Body temperature, (l) body weight, (m) clinical score, and (n) survival rate changes in septic mice with different treatments (n = 7). (o‐r) UREA, ALT, AST, and CREA in the serum of septic mice with different treatments. (s–v) Serum levels of CRP, TNF‐α, IL‐6, and TGF‐β in septic mice with different treatments (n = 3). Antibiotic cocktail (vancomycin (50 µg/mL) + colistin (50 µg/mL)). Data are presented as mean ± SD. Group differences were analyzed by one‐way ANOVA followed by Tukey's HSD post‐hoc test. Significance is denoted as ^*^ *p* < 0.05, ^**^ *p* < 0.01, ^***^ *p* < 0.001, and ns indicates no significant difference (*p* ≥ 0.05).

Since MICP@HG exhibited pronounced infection‐site targeting capability in vitro, in vivo biodistribution and infection‐targeting behavior were further evaluated using a muscle infection mouse model induced by *E. asburiae* and MRSA (Figure 7a). We detected a pronounced fluorescence signal at the infection site in the MICP@HG‐treated group at 24 h after intravenous administration of MI, MICP, or MICP@HG at 0.5 h post infection, whereas we observed minimal fluorescence accumulation in the MI or MICP groups (Figure 7b). Notably, time‐dependent imaging revealed that MICP@HG fluorescence appeared as early as 3 h post injection and progressively intensified from 3 to 24 h, consistent with progressive infection‐site accumulation and confirming its infection‐site–targeting capability (Figure 7c). This targeting property facilitates efficient nanozyme accumulation from systemic circulation to infection sites. The therapeutic efficacy of MICP@HG was further evaluated in a polymicrobial sepsis mouse model, which recapitulates clinically relevant polymicrobial sepsis caused by mixed bacterial infections. A combination of MRSA and *E. asburiae*—two multidrug‐resistant pathogens identified as “serious threats” by the Centers for Disease Control and Prevention—was administered intraperitoneally to induce systemic infection. In this model, bacterial colony‐forming units (CFUs) were quantified in peritoneal lavage fluid and major organs 24 h after infection (Figure 7d). MICP@HG treatment markedly reduced bacterial burden in both the peritoneal cavity and major organs, exhibiting markedly enhanced bacterial clearance efficacy compared with the control, MI and MICP groups (Figure 7e–h; Figure S9). This pronounced therapeutic effect indicates that MICP@HG effectively accumulates at infection sites and exerts potent in vivo antibacterial activity, demonstrating its potential for treating peritonitis‐induced systemic infections. Representative anatomical images further confirmed these observations (Figure 7i). Mice in the untreated sepsis group displayed extensive purulent exudation, diffuse hemorrhage, and pronounced intestinal wall swelling, indicative of acute peritonitis. In contrast, MICP@HG‐treated mice showed significantly reduced inflammation, lighter exudate coloration, and preservation of intestinal morphology, suggesting effective infection control and inflammatory resolution. This therapeutic outcome is attributed to the coordinated antibacterial and immunoregulatory activities of MICP@HG, which collectively suppress bacterial proliferation, mitigate inflammatory injury, and promote tissue recovery. The systemic therapeutic efficacy of MICP@HG was further evaluated by monitoring the physiological and pathological parameters of septic mice over a 7‐day period (Figure 7j). Mice in the untreated sepsis group exhibited rapid systemic deterioration, including a sharp drop in body temperature, significant weight loss, and worsening clinical scores. In contrast, MICP@HG treatment effectively stabilized body temperature, mitigated weight loss, and improved clinical scores throughout the observation period. Remarkably, all mice in the MICP@HG group survived during the 7‐day period, maintaining complete survival throughout the observation period, with a survival rate significantly higher than that of the untreated group (Figure 7k–n). Collectively, MICP@HG confers substantial protection against polymicrobial sepsis by efficiently eradicating bacteria, alleviating systemic inflammation, and promoting systemic physiological recovery.

The therapeutic efficacy of MICP@HG in preventing multiple organ failure, a major determinant of sepsis‐associated mortality, was further assessed through blood biochemical analysis. As shown in Figure 7o–r, serum biomarkers indicative of liver injury (alanine aminotransferase, ALT; aspartate aminotransferase, AST) and renal dysfunction (UREA; creatinine, CREA) were markedly elevated following bacterial inoculation, confirming severe multiorgan injury in the untreated sepsis group. In contrast, MICP@HG treatment significantly reduced these biomarkers to levels approaching physiological ranges, demonstrating effective protection of hepatic and renal functions against infection‐induced injury. MICP treatment provided only partial alleviation, whereas MI, lacking antibacterial activity, failed to confer any observable benefit. These results highlight that MICP@HG protects against sepsis‐associated organ dysfunction and improves overall survival outcomes in septic mice. Routine hematological analysis revealed a significant increase in neutrophils (Neu) and white blood cells (WBCs) in septic mice, indicating severe systemic inflammatory activation (Figure S10). Following MICP@HG treatment, Neu and WBCs returned toward normal levels, suggesting effective suppression of infection‐induced excessive inflammatory activation. In addition, the concentration of C‐reactive protein (CRP)‐an early and sensitive biomarker of sepsis—was markedly elevated in the model group but significantly reduced after MICP@HG treatment (Figure 7s). Consistent with these findings, ELISA showed that the serum levels of pro‐inflammatory cytokines IL‐6 and TNF‐α were substantially decreased and restored toward baseline levels following MICP@HG treatment, whereas the level of the anti‐inflammatory cytokine TGF‐β was significantly increased (Figure 7t–v). Collectively, MICP@HG effectively alleviates systemic inflammation, restores systemic inflammatory balance, and prevents immune overactivation in septic mice. The coordinated antibacterial, antioxidative, and immunoregulatory activities of MICP@HG underpin its potent therapeutic efficacy in polymicrobial sepsis.

### 2.8 In Vivo Antioxidative, Anti‐Inflammatory, and Immunomodulatory Effects of MICP@HG

**FIGURE 8:**
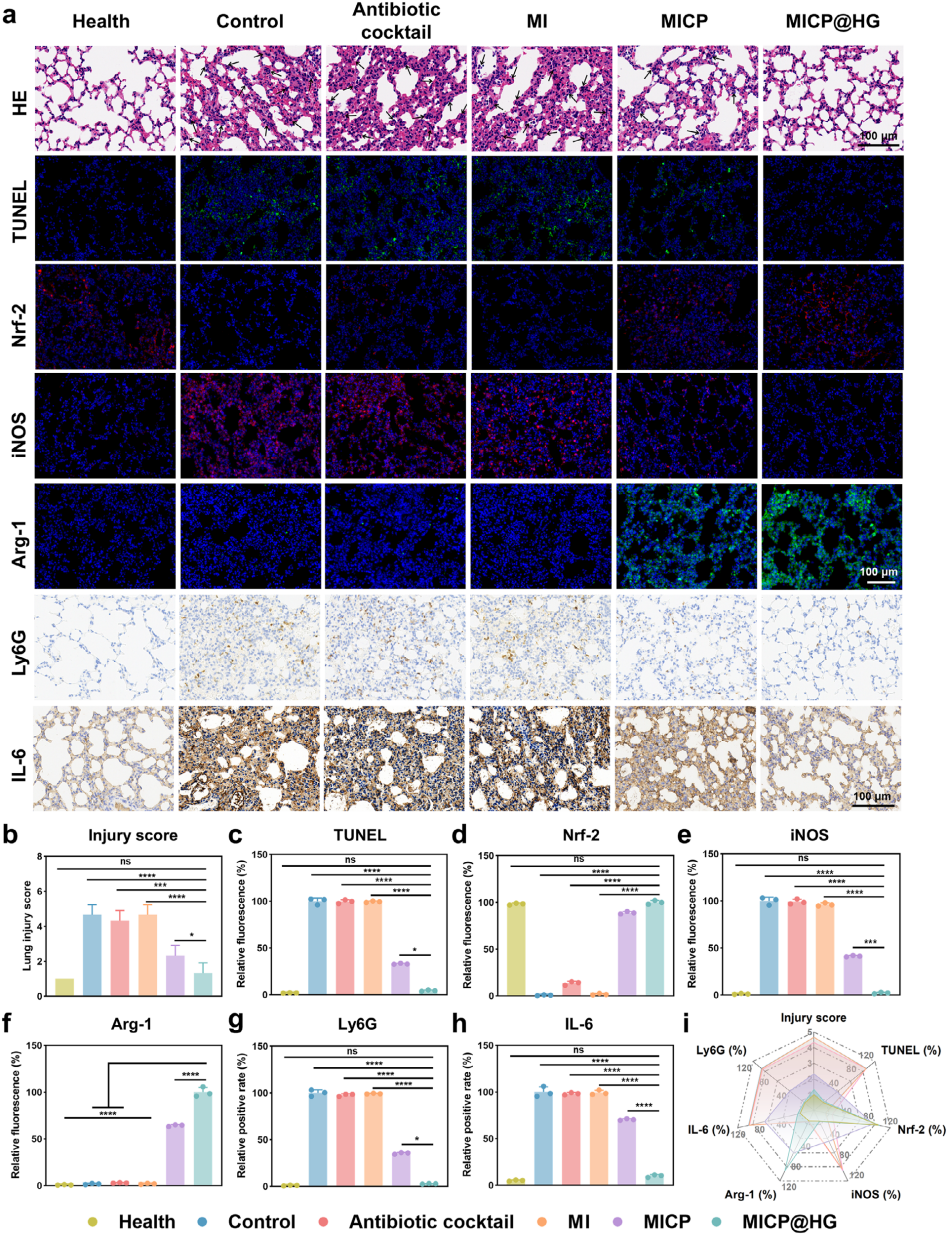
Antioxidant, anti‐inflammatory and immunomodulatory effects of MICP@HG in vivo. (a) Representative images of lung tissue sections from infected mice under different treatments, showing HE staining, immunofluorescence staining of TUNEL, Nrf‐2, iNOS, and Arg‐1, as well as immunohistochemical staining of IL‐6 and Ly6G. (b–h) Quantitative analysis of (b) lung injury scores and expression levels of (c) TUNEL, (d) Nrf‐2, (e) iNOS, (f) Arg‐1, (g) Ly6G, and (h) IL‐6 in lung tissues from infected mice across the treatment groups. (i) Radar chart providing a consolidated view of all quantitative metrics. Antibiotic cocktail (vancomycin (50 µg/mL) + colistin (50 µg/mL)). Data are presented as mean ± SD, n = 3. Group differences were analyzed by one‐way ANOVA followed by Tukey's HSD post‐hoc test. Significance is denoted as *p < 0.05, **p < 0.01, ***p < 0.001, and ns indicates no significant difference (*p* ≥ 0.05).

Given that multiorgan dysfunction is a major cause of mortality in severe sepsis [60], histopathological and tissue‐level analyses were conducted 24 h after treatment. Hematoxylin and eosin (H&E) staining of the liver, lung, and kidneys from septic mice revealed extensive inflammatory infiltration, thickened inter‐alveolar septa, and tubular epithelial swelling, representing characteristic pathological features of sepsis‐associated multiorgan injury (Figure 8a,b; Figures S11a,b and S12a,b). Administration of MICP@HG markedly alleviated these pathological changes and reduced tissue injury scores, consistent with improved survival outcomes. To elucidate the mechanisms underlying these histological improvements, oxidative stress– and apoptosis‐associated markers were assessed in vivo. TdT‐mediated dUTP‐nick end labeling (TUNEL) staining demonstrated a marked reduction in apoptotic cells in MICP@HG‐treated mice compared with controls, confirming its pronounced antioxidative and cytoprotective effects in multiple organs (Figure 8a,c; Figures S11a,c and S12a,c). Consistently, immunofluorescence staining showed that the expression of the oxidative stress‐responsive transcription factor Nrf‐2 was extremely low in the untreated and MI groups, whereas MICP@HG treatment significantly upregulated Nrf‐2 expression across liver, lung, and kidney tissues (Figure 8a,d; Figures S11a,d and S12a,d) [61, 62, 63]. Macrophage‐mediated inflammatory progression is highly correlated with organ injury [64]. Further immunofluorescence analysis of macrophage markers revealed that MICP@HG markedly promoted macrophage immune reprogramming toward an anti‐inflammatory phenotype. The fluorescence intensity of the pro‐inflammatory marker iNOS was reduced, whereas that of the anti‐inflammatory marker Arg‐1 was elevated in the MICP@HG group compared with the PBS, antibiotic‐cocktail, and MI groups (Figure 8a,e,f; Figures S11a,e,f and S12a,e,f). These findings indicate that MICP@HG reprograms macrophages from a pro‐inflammatory (M1) to a reparative (M2) phenotype.

In septic mice, extensive neutrophil infiltration was observed in the liver, lung, and kidney, accompanied by widespread tissue degeneration. Immunohistochemical staining of Ly6G, a neutrophil marker, showed strong positive signals in the PBS, antibiotic‐cocktail, and MI groups, whereas Ly6G expression in MICP@HG‐treated mice was largely restored toward physiological levels (Figure 8a,g; Figures S11a,g and S12a,g). To further assess inflammatory burden, the expression levels of IL‐6 in liver, lung, and kidney tissues were examined by IHC. IL‐6 expression was downregulated after MICP@HG treatment, whereas its levels remained high in the PBS, antibiotic‐cocktail, and MI groups (Figure 8a,h; Figures S11a,h and S12a,h). Collectively, MICP@HG effectively mitigates sepsis‐induced multi‐organ injury through coordinated antioxidative, cytoprotective, and immunoregulatory activities, ultimately restoring inflammatory tissue homeostasis and suppressing systemic inflammation (Figure 8i; Figures S11i and S12i).

### 2.9 Biosafety and Prophylactic Effects of the MICP@HG In Vivo

**FIGURE 9:**
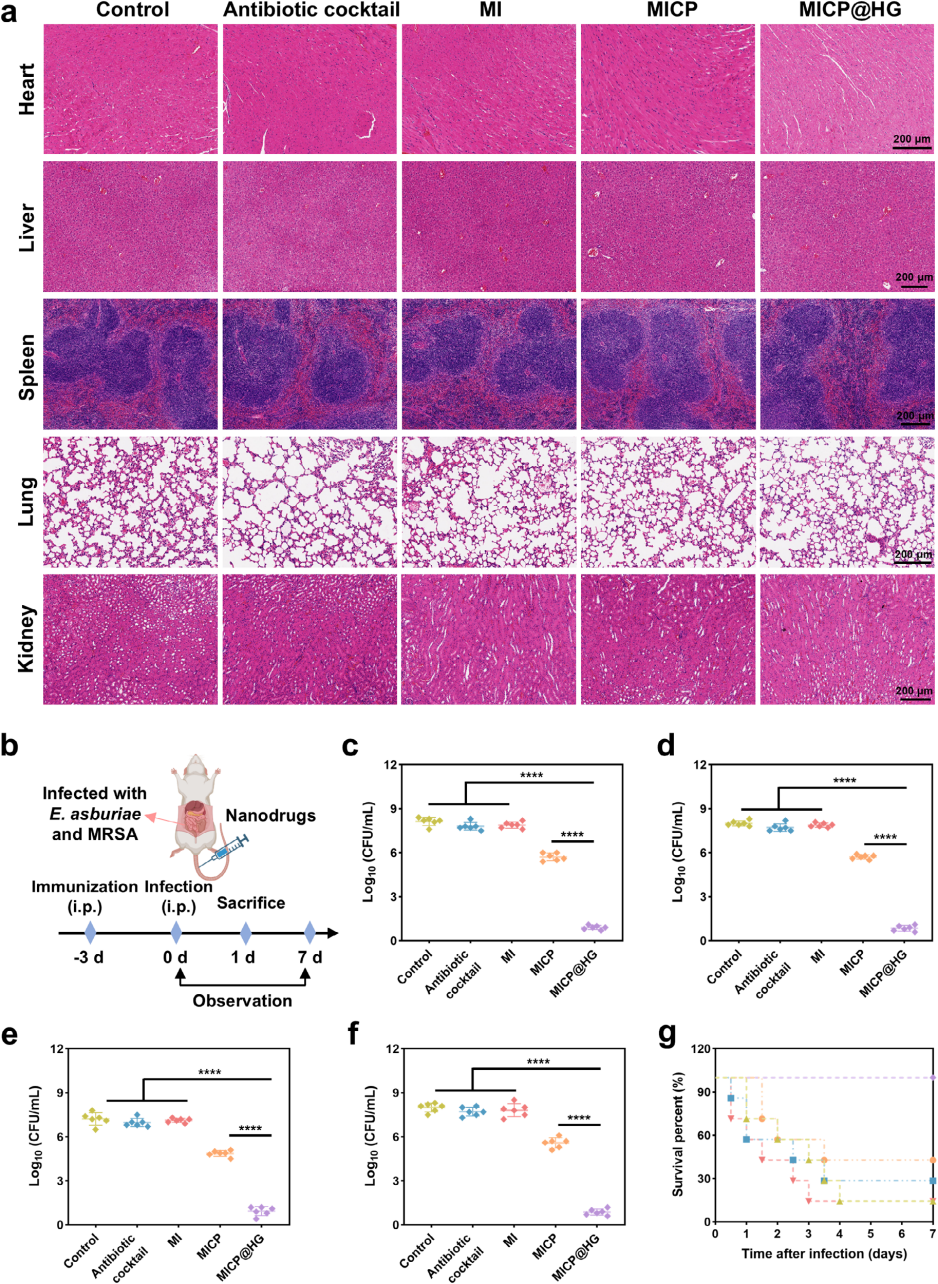
Biosafety and prophylactic effects of MICP@HG in vivo. (a) Histological evaluation of heart, liver, spleen, lung, and kidney by H&E staining after different treatments. (b) Schematics of the prophylactic experiment design of a single dose of MICP@HG (100 µg/mL). Quantification of the number of bacteria in (c) peritoneal fluid, (d) liver, (e) lung, and (f) kidney of the infected mice after different treatments. Infected mice with PBS were used as a control (n = 6). (g) Survival curves of the mice intraperitoneally infected with *E. asburiae* and MRSA after different treatments (n = 7). Antibiotic cocktail (vancomycin (50 µg/mL) + colistin (50 µg/mL)). Data are presented as mean ± SD. Group differences were analyzed by one‐way ANOVA followed by Tukey's HSD post‐hoc test. Significance is denoted as ^*^ *p* < 0.05, ^**^ *p* < 0.01, ^***^ *p* < 0.001, and ns indicates no significant difference (*p* ≥ 0.05).

To evaluate the translational potential of MICP@HG, in vivo biosafety was comprehensively evaluated following intravenous administration in mice. Different formulations were injected via the tail vein, and physiological parameters and histological structures of major organs were examined on day 14 post injection. Routine hematological parameters showed no significant differences among groups, indicating minimal hematological toxicity (Figure S13). Similarly, serum biochemical analyses revealed that the levels of hepatic function markers (ALT, AST, ALP) and renal function indicators (UREA, CREA) in MICP@HG‐treated mice were comparable to those in the PBS control group, indicating negligible hepatic and renal toxicity (Figure S14). In addition, a hemolysis assay was performed to evaluate the hemocompatibility of MI, MICP, and MICP@HG. No visible hemolysis was observed in any group, and the hemolysis rates of MICP@HG remained below 3% at all tested concentrations, demonstrating excellent blood compatibility (Figure S15). Histological evaluation further supported these findings. H&E staining of the liver, kidney, heart, spleen, and lung showed no evidence of inflammatory infiltration, fibrosis, or necrosis in MICP@HG‐treated mice compared with the control group (Figure 9a). Furthermore, in vivo and ex vivo fluorescence imaging revealed transient accumulation of MICP@HG in the liver, lung, and kidney within the first 3 days post injection, followed by gradual clearance over days 1–6. No detectable fluorescence signal was observed in any organ after day 7, indicating effective systemic clearance and biodegradability of the nanoplatform. The combination of low systemic toxicity, rapid metabolic clearance, and potent therapeutic efficacy underscores the translational potential of MICP@HG as a safe and biodegradable nanotherapeutic for severe infectious diseases (Figure S16).

After confirming the therapeutic efficacy and biocompatibility of MICP@HG in vivo, we evaluated its prophylactic immunoprotective potential against polymicrobial infection. Mice were subcutaneously immunized with MICP@HG and subsequently challenged intraperitoneally with *E. asburiae* and MRSA three days post immunization (Figure 9b). Bacterial burden analyses revealed that the PBS control group exhibited minimal bacterial reduction, whereas mice immunized with MICP@HG displayed markedly decreased bacterial counts in the peritoneal fluid, liver, lung, and kidney, corresponding to approximately 5–7 log reductions in CFUs (Figure 9c–f; Figure S17). These results indicate that MICP@HG pre‐immunization significantly enhanced systemic antibacterial protection. Survival monitoring further confirmed the strong prophylactic efficacy of MICP@HG. All mice in the MICP@HG‐immunized group survived the 7‐day infection period, maintaining complete survival throughout the observation period, whereas control mice exhibited rapid deterioration and high mortality (Figure 9g). The improved survival outcome is attributed to the innate immune activation induced by MICP@HG, which functionally primes macrophages and other innate immune cells for enhanced bacterial recognition and clearance across multiple organs. This vaccine‐like innate immune priming effect demonstrates that MICP@HG not only serves as a therapeutic nanoplatform but also functions as a vaccine‐like immunomodulator capable of establishing prophylactic immunoprotection against subsequent polymicrobial infections.

## 3 Discussion

In summary, we developed a microenvironment‐adaptive theranostic nanozyme platform, MICP@HG, that integrates infection imaging, catalytic bactericidal activity, and immune modulation for the treatment of polymicrobial sepsis. By combining a Cu‐polyphenol catalytic shell with a targeting and immunoregulatory polysaccharide interface, MICP@HG enables coordinated antibacterial and host‐regulatory activities within a single nanosystem. Importantly, this study establishes a transition from static nanotherapeutics toward disease‐stage synchronized therapeutic strategies capable of dynamically responding to the evolving septic microenvironment.

Unlike conventional static or constitutively active therapeutic systems, MICP@HG dynamically reconfigures its catalytic and immunological activities in response to microenvironmental cues, thereby addressing the dynamic interplay among pathogen burden, oxidative stress, inflammatory injury, and immune dysregulation during sepsis progression. Through acidic microenvironment‐triggered ROS amplification, copper‐associated metabolic collapse, antioxidative regulation, and macrophage immune reprogramming, MICP@HG achieves coordinated regulation of infection clearance and host recovery. The functional synchronization between catalytic bactericidal activity and host‐directed immunomodulation highlights a therapeutic framework in which antimicrobial intervention and immune restoration are mechanistically integrated.

Beyond the current study, future investigations should focus on systematically characterizing the spatiotemporal activation behavior of nanozyme functions in vivo and further quantitatively separating of HA‐ and β‐glucan‐specific contributions in vivo. Extending this disease‐stage synchronized therapeutic design to other dynamic inflammatory and infectious disorders may provide a broadly applicable framework for adaptive nanomedicine. In addition, integrating MICP@HG with existing standard‐of‐care therapies and evaluating the durability of its innate immune priming effects will be important steps toward clinical translation [65]. Collectively, this work establishes a conceptual framework for programmable disease‐stage nanotherapy that integrates catalytic regulation with immune adaptation. By synchronizing therapeutic function with pathological progression, this strategy provides a foundation for next‐generation therapeutic approaches targeting complex pathophysiological disorders such as sepsis.

Although the present study demonstrates pH‐responsive catalytic switching in vitro and confirms the therapeutic efficacy of MICP@HG in polymicrobial sepsis models, the spatiotemporal activation of its distinct nanozyme functions in vivo remains to be fully elucidated. In particular, future studies should perform time‐resolved analyses across defined stages of sepsis, including early infection, inflammatory progression, and recovery, to dynamically monitor bacterial burden, local pH evolution, ROS homeostasis, inflammatory cytokines, macrophage polarization, and organ injury. Such investigations will further clarify the in vivo stage‐dependent therapeutic behavior of MICP@HG and support its translational development as a disease‐stage synchronized nanotherapeutic platform.

## 4 Experimental Section

### 4.1 Ethics Statement

Animal experiments were carried out with the approval of the Laboratory Animal Center of Hangzhou Medical College (Approval No. ZJCLA‐IACUC‐20011394). All animal procedures were performed in accordance with the institutional guidelines for the care and use of laboratory animals and complied with all relevant national regulations.

### 4.2 Statistical Analysis

Results are presented as mean ± SD. Mice were randomly assigned to treatment groups, and fields for microscopic analysis were selected at random. Differences between the experimental groups were assessed using one‐way analysis of variance (ANOVA) followed by Tukey's post hoc comparison test (^*^ *p* < 0.05; ^**^ *p* < 0.01; ^***^ *p* < 0.001; ^****^ *p* <0.0001). Survival analysis was determined using the log‐rank test. Experiments were repeated multiple times as independent experiments, as indicated in the figure captions. Analyses were performed using GraphPad Prism software and Origin Pro 2023. *p *< 0.05 was considered statistically significant.

## Author Contributions

**A. Z**.: conceptualization, methodology, investigation, data curation, and Writing – original draft. **X. Z**.: methodology, validation, formal analysis, Writing – review and editing. **R. Z**.: visualization. **C. X**.: investigation. **Y. Z**.: validation. **M. L**.: investigation. **T. Y**.: methodology. **Y. G**.: validation. **Z. R**.: formal analysis. **Y. F**.: formal analysis. **Y. X**.: Resources, supervision, validation, and Writing – review and editing. **J. Z**.: Resources, supervision, validation, and Writing – review and editing.

## Conflicts of Interest

The authors declare no conflicts of interest

## Supporting information




**Supporting File**: advs77231‐sup‐0001‐SuppMat.docx.

## Data Availability

The data that support the findings of this study are openly available in China National Center for Bioinformation at https://ngdc.cncb.ac.cn/gsub/submit/gsa/list, reference number GSA: CRA047367.
