# Supplementary material for: Disease‐Stage Synchronized Nanozyme Therapy for Polymicrobial Sepsis through Adaptive Catalytic and Immune Reprogramming
Source: Adv Sci (Weinh). 2026 Sep 8:e77231. Online ahead of print. doi: 10.1002/advs.77231 (PMC13554492; doi:10.1002/advs.77231)
Supplement: Supplementary file 1 [file ADVS-9999-e77231-s001.docx]

**Disease-stage synchronized nanozyme therapy for polymicrobial sepsis through adaptive catalytic and immune reprogramming**

Anlai Zou, ^1,2^ Xiaoxue Zhu, ^2,3^ Runhan Zhu, ^1,2^ Chengchu Xue, ^1,2^ Qinyu Zhang, ^1,2^ Mengke Li, ^2,3^ Ting Yu, ^1,2^ Yuzhen Gao, ^1,2^ Zhi Ruan, ^1,2^ Ying Fu, ^1,2^ Yunlei Xianyu, ^1,2,3,*^ and Jun Zhang ^1,2,*^

^1^Department of Clinical Laboratory, Sir Run Run Shaw Hospital of Zhejiang University School of Medicine, Hangzhou 310016, Zhejiang, China

^2^Key Laboratory of Precision Medicine in Diagnosis and Monitoring Research of Zhejiang Province, Hangzhou 310016, Zhejiang, China

^3^College of Biosystems Engineering and Food Science, Zhejiang Key Laboratory of Agro-Food Resources and High-Value Utilization, Zhejiang University, Hangzhou, 310058, China

^*^Correspondence: Yunlei Xianyu (xianyu19@zju.edu.cn), Jun Zhang (jameszhang2000@zju.edu.cn)

**Content List**

[**Experimental section	S4**](#_Toc234999594)

[**Materials and agents	S4**](#_Toc234999595)

[**Synthesis of MSN	S4**](#_Toc234999596)

[**Synthesis of MI	S5**](#_Toc234999597)

[**Synthesis of MICP	S5**](#_Toc234999598)

[**Synthesis of MICP@HG	S5**](#_Toc234999599)

[**Characterization of the prepared MSNs, MI, MICP and MICP@HG	S5**](#_Toc234999600)

[**Peroxidase (POD)-like catalytic activity of MICP@HG	S6**](#_Toc234999601)

[**SOD-like activity of MICP@HG	S6**](#_Toc234999602)

[**CAT-like activity of MICP@HG	S7**](#_Toc234999603)

[**GPx-like activity of MICP@HG	S7**](#_Toc234999604)

[**In vitro antibacterial activity assay	S7**](#_Toc234999605)

[**Live/dead fluorescent staining	S8**](#_Toc234999606)

[**Bacterial morphology observation	S8**](#_Toc234999607)

[**Cuproptosis-like death	S9**](#_Toc234999608)

[**Cell compatibility	S9**](#_Toc234999609)

[**Intracellular ROS detection	S10**](#_Toc234999610)

[**Macrophage polarization in vitro	S10**](#_Toc234999611)

[**Transwell chemotaxis assay	S10**](#_Toc234999612)

[**Hemolysis assay	S11**](#_Toc234999613)

[**In vivo biocompatibility of MICP@HG	S11**](#_Toc234999614)

[**In vivo imaging of MICP@HG in mouse bacterial infection models	S11**](#_Toc234999615)

[**MRSA and *E. asburiae*-induced polymicrobial peritonitis infection model	S11**](#_Toc234999616)

[**CFU detection	S12**](#_Toc234999617)

[**Biochemical parameters, inflammatory cytokines, and histopathological analysis	S12**](#_Toc234999618)

[**In vivo prophylactic effect against bacterial infection	S13**](#_Toc234999619)

[**RNA-seq analysis	S14**](#_Toc234999620)

[**Supporting figure	S15**](#_Toc234999621)

[**Figure S1	S15**](#_Toc234999622)

[**Figure S2	S16**](#_Toc234999623)

[**Figure S3.	S17**](#_Toc234999624)

[**Figure S4	S18**](#_Toc234999625)

[**Figure S5	S19**](#_Toc234999626)

[**Figure S6	S20**](#_Toc234999627)

[**Figure S7.	S21**](#_Toc234999628)

[**Figure S8	S22**](#_Toc234999629)

[**Figure S9	S23**](#_Toc234999630)

[**Figure S10	S24**](#_Toc234999631)

[**Figure S11	S25**](#_Toc234999632)

[**Figure S12	S26**](#_Toc234999633)

[**Figure S13	S27**](#_Toc234999634)

[**Figure S14	S28**](#_Toc234999635)

[**Figure S15	S29**](#_Toc234999636)

[**Figure S16	S30**](#_Toc234999637)

[**Figure S17	S31**](#_Toc234999638)

[**References	S32**](#_Toc234999639)

Experimental section

**Materials and agents**

Triethanolamine (TEA), IR-780, and cetyl trimethyl ammonium bromide (CTAB), cupric chloride dihydrate (CuCl_2_·2H_2_O), piceatannol (Pic), polyvinylpyrrolidone K30 (PVP K30), rhodamine B, glutathione (GSH), 2,9-dimethyl-1,10-phenanthroline, 5,5’-dithiobis(2-nitrobenzoic acid) (DTNB) and L-methionine were purchased from Aldrich. Hyaluronic acid (HA), sodium salicylate (NaSal), tetraethyl orthosilicate (TEOS), 3,3′,5,5′-tetramethylbenzidineand (TMB), nitroblue tetrazolium (NBT), and riboflavin were purchased from Macklin Biochemical Co., Ltd (Shanghai, China). APC anti-mouse CD206 (141707), PE anti-mouse CD86 (105007) were purchased from BioLegend. H&E staining solution and dialysis bags were purchased from Sangon Biotech Co., Ltd (Shanghai, China). Reactive oxygen species (ROS) assay kit, malondialdehyde (MDA) colorimetric assay kit, cell counting kit-8 (CCK-8), calcein AM/PI double staining kit, ATP assay kit, and immunostaining fixative solution were purchased from Beyotime Biotech Co., Ltd (Shanghai, China). The plasmid extraction kit was purchased from TIANGEN Biotech Co., Ltd (Beijing, China). All bacterial culture reagents (LB medium) were obtained from Beijing Solarbio Science & Technology Co., Ltd (China). TUNEL apoptosis assay kit was sourced from Solarbio Science & Technology (Beijing, China).

## Synthesis of MSN

Triethanolamine (TEA) was dispersed in 25 mL of deionized water and stirred at 80 °C for 30 min. Subsequently, 0.38 g of CTAB and 0.168 g of sodium salicylate were added to the mixture, and the resulting mixture was stirred at 1000-1500 rpm for 1 h. Thereafter, TEOS and BTSE were added, and the mixture was stirred for 12 h. The resulting product was washed twice with ethanol and then redispersed in 30 mL of ethanol. For CTAB removal, a solution containing 200 mL of ethanol and 400 μL of hydrochloric acid was prepared at 60 °C in a glass bottle. The sample was then subjected to this acid-ethanol treatment three times for 3, 3, and 12 h, respectively [1].

## Synthesis of MI

IR-780 was co-loaded into MSNs through a simple adsorption process to prepare MI NPs. First, 10 mg of MSNs was dispersed in 1 mL of methanol. Then, 2 mg of IR-780 was dissolved in 1 mL of methanol and subsequently mixed with MSNs under continuous stirring for 3 d at room temperature. The mixture was centrifuged at 15000 rpm and washed with methanol to obtain MI NPs. Meanwhile, the supernatant was collected for further determination of the loading contents of IR-780 in MI NPs using an Infinite 200Pro microplate reader (TECAN, Switzerland) at 780 nm.

## Synthesis of MICP

First, 30 mg of MI and 85 mg of CuCl_2_·2H_2_O were mixed with 200 mg of PVP K30 in 40 mL of deionized water and stirred vigorously for 1 h, followed by the slow addition of a piceatannol methanol solution (1 mL, 60 mg/mL). Finally, the resulting mixture was adjusted to pH 12 with NaOH solution (8 mg/mL), stirred continuously for 24 h, and centrifuged at 12 000 rpm for 15 min to obtain a brown solid product [2].

## Synthesis of MICP@HG

5 mg of MICP nanoparticles were dispersed in 5 mL of deionized water. Afterwards, 1 mL of HA (2.5 mg/mL) and β-glucan (2.5 mg/mL) were added and stirred for 12 h. Finally, the resulting mixture was centrifuged at 12 000 rpm for 15 min and washed it repeatedly with deionized water three times.

## Characterization of the prepared MSNs, MI, MICP and MICP@HG

Transmission electron microscopy (TEM) images were obtained using a JEOL 2100Plus TEM operated at an accelerating voltage of 120 kV. Scanning electron microscopy (SEM) images were acquired using the GeminiSEM 300 (Zeiss, Germany). A Zetasizer Nano ZS90 (Malvern Instruments, UK) was used to measure particle size and zeta potential. The crystal structure was analyzed using an X-ray powder diffractometer (XRD, Rigaku SmartLab SE, Japan) with high-intensity Cu Kα radiation. The chemical composition and bonding states were determined using X-ray photoelectron spectroscopy (XPS, Thermo Scientific K-Alpha, USA) and Fourier-transform infrared spectroscopy (FT-IR, Thermo Scientific Nicolet iS20, USA). UV-Vis spectra were recorded using an Infinite 200Pro microplate reader (TECAN, Switzerland). The surface area and pore size of MSNs were obtained from the nitrogen adsorption-desorption isotherms determined using an ASAP2020 specific surface area and aperture analyzer (Micromeritics, Norcross, GA, USA). The elemental distribution in MICP@HG was analyzed using energy-dispersive X-ray spectroscopy (EDS) mapping on a JEM-2100F instrument (JEOL, Tokyo, Japan).

## Peroxidase (POD)-like catalytic activity of MICP@HG

The color change of POD-like MICP@HG toward 3,3’,5,5’-tetramethylbenzidine (TMB) was photographed at room temperature after 10 min of coincubation. The steady-state kinetic assay of MICP@HG toward TMB in the presence of H_2_O_2_ was carried out at room temperature. The final concentrations of TMB, H_2_O_2_, and MICP@HG were 10 mg/mL, 1 mM, and 100 μg/mL, respectively. The reaction time was 10 minutes. The buffer solution was 0.2 M NaAc-HAc. The pH values were adjusted between 3.5 and 9.5 by mixing different volumes of NaOH and HAc. The reactions were monitored at 652 nm in time-scan mode using a microplate reader. The kinetic parameters were obtained using linear fitting of the Lineweaver–Burk double-reciprocal plot derived from the Michaelis–Menten equation:

$$\frac{\text{1}}{v}\text{ =}\frac{\text{K}\text{m}}{\text{V}\text{max}}\text{ × }\frac{\text{1}}{[S]}+ \frac{\text{1}}{\text{V}\text{max}}$$

where $v$ is the initial velocity, V_max_ is the maximal reaction velocity, [S] is the concentration of substrate, and K_m_ is the Michaelis constant.

## SOD-like activity of MICP@HG

0.3 mL of methionine (100 mM), 0.3 mL of riboflavin (200 µM) and 0.3 mL of NBT (800 µM) were mixed in PBS (pH 3.5-9.5) with MICP@HG at final concentrations of 25, 50, 100, 150 and 200 µg/mL, respectively. Then, the solution was irradiated with a UV lamp for 10 min, and the absorbance at 560 nm was measured immediately after irradiation using a microplate reader. The inhibition rate was computed as follows:

Scavenging rate (%)=(𝐴_0_−𝐴)/𝐴_0_×100	(1)

where *A*_0_ refers to the absorbance of the control group and *A* refers to the absorbance of the experimental samples. Three independent samples were analyzed for each group, and the results are presented as the mean ± standard deviation (SD).

## CAT-like activity of MICP@HG

The catalase-like (CAT-like) activity was assessed using [Ru(dpp)₃]Cl₂, a luminescent oxygen sensor whose fluorescence is quenched by O₂. Specifically, MICP@HG (10 μL, 0.75-6 mg/mL), H_2_O_2_ (20 μL, 100 mM), and [Ru(dpp)₃]Cl₂ (20 μL, 8.5 μM) were added to PBS (250 μL, pH 7.5). After 15 min of incubation, O_2_ generation was monitored by recording fluorescence intensity changes (excitation at 488 nm, emission at 624 nm). The pH dependence of the CAT-like activity of MICP@HG was also investigated. Based on the Michaelis-Menten equation, the kinetic parameters were calculated from the Lineweaver-Burk plot as described above.

## GPx-like activity of MICP@HG

GSH oxidation was detected via the Ellman method, and all the experiments were conducted in a dark environment. The yellow product, 2-nitro-5-thiobenzoic acid (TNB), was synthesized via the reaction of Ellman's reagent (5, 5’-dithio-bis-(2-nitrobenzoic acid), DTNB) and thiol groups (-SH) in GSH [3]. Therefore, DTNB was used as a substrate to evaluate the GPx-like activity of MICP@HG. Specifically, 50 µL of DTNB (1.5 mg/mL), 50 µL of GSH (1 mg/mL), 2 mL of PBS and MICP@HG were added to the centrifuge tube. The final concentrations were 0, 50, 100, 150, and 200 µg/mL, respectively. After 10 min of incubation, the characteristic absorbance was measured using a microplate reader.

## In vitro antibacterial activity assay

The *Enterobacter asburiae* (*E. asburiae*) strain co-harboring the *mcr-10* and *bla*_NDM-1_ genes was a clinical colistin-resistant isolate. Matrix-assisted laser desorption/ionization time-of-flight (MALDI-TOF) mass spectrometry (bioMérieux, France) was used for species identification. Minimum inhibitory concentration (MIC) values of the *E. asburiae* were determined by the standard broth microdilution method, following the CLSI 2025 guidelines [4,5]. Briefly, antibacterial agents were two-fold serially diluted in LB medium and mixed with an equal volume of bacterial suspensions containing approximately 10^6^ CFU/mL in a UV-sterilized 96-well microtiter plate. After incubation at 37 °C for 24 h, MIC values were defined as the lowest concentration of antibacterial agents that completely inhibited visible bacterial growth.

Bacterial strains were cultured in lysogeny broth (LB) medium at 37 °C under shaking conditions until the logarithmic growth phase. The concentration of the bacteria was determined by measuring the optical density at 600 nm (OD_600_) using a microplate reader. Bacterial suspensions were divided into 8 groups: Control, MI, MICP, MICP@HG, H_2_O_2_, MI/H_2_O_2_, MICP/H_2_O_2_, and MICP@HG/ H_2_O_2_. The spread plate method was used to investigate the antibacterial activity. The samples were mixed with bacterial suspensions (10^8^ CFU/mL). The mixtures were spread on LB agar plates and cultured for 6 h, after which the resulting colonies were counted. Each experiment was repeated at least three times.

## Live/dead fluorescent staining

Bacterial cultures were washed three times with PBS, centrifuged at 6000 rpm for 5 min, and then serially diluted. Bacterial suspensions (10^6^ CFU/mL) were incubated with 10 μL of MICP@HG or MICP@HG/H_2_O_2_, followed by staining with a mixture of 10 μL Calcein-AM and PI for 20 min in the dark. After being washed three times with PBS, the stained bacterial suspensions were transferred to slides and observed under a laser scanning confocal microscope (CLSM). Bacterial samples treated with PBS alone served as control group.

## Bacterial morphology observation

A bacterial suspension (1 mL, 1 × 10^8^ CFU/mL) and samples (100 µg/mL) were incubated for 6 h. Then the bacteria were fixed with 2.5% glutaraldehyde and dehydrated through a graded ethanol series. The bacterial morphology was examined by SEM and TEM.

## Cuproptosis-like death

The *E. asburiae*, CREC, and MRSA were treated with different formulations. The bacterial cell suspensions were filtered using a 0.22 µm syringe filter. The absorbance at 260 nm (OD_260_) of the filtrates was then measured using a microplate reader to assess nucleic acid leakage. Subsequently, the protein concentration in the supernatant was measured using a BCA protein assay kit. Bacterial membrane potential was assessed using the voltage-sensitive dye DiBAC₄(3), and fluorescence images were captured by a confocal laser scanning microscopy to evaluate membrane depolarization.

The level of ROS in bacteria was detected by a DCFH-DA probe. MDA detection kit (Beyotime) was used to detect the level of bacterial lipid peroxidation, and GSH/GSSG detection kit (Beyotime) was used to detect the GSH/GSSG ratio. In addition, the effects of MI, MICP, and MICP@HG on bacterial respiratory chain complexes II/IV were studied by using the Succinate Dehydrogenase Assay Kit and Cytochrome C Oxidase Assay Kit (Beyotime). The intracellular Cu content in bacteria was detected by a Cu detection kit (Elabscience). Pyruvate detection kit (Beyotime) was used to detect pyruvate accumulation in bacteria. ATP assay kit (Beyotime) was used to detect ATP level in bacteria. To study the effect of a cuproptosis-like death inhibitor on the antibacterial activity, bacteria were incubated with EDTA and *E. asburiae*, MRSA, CREC at 37 °C for 24 h, and the bacterial viability was determined by the spread plate method. To evaluate the extent of bacterial plasmid damage induced by various samples, a plasmid extraction kit from TIANGEN was employed. The resulting DNA fragments were quantitatively analyzed by agarose gel electrophoresis.

## Cell compatibility

The cytotoxicity of MICP@HG on HUVEC cells and RAW 264.7 macrophage cells was investigated. Briefly, HUVEC cells and RAW 264.7 macrophage cells (5000 cells per well) were inoculated into 96-well plates and incubated overnight. Subsequently, MICP@HG (0, 50, 100, 150, 200 and 300 μg/mL) was added to the plates. After incubation for an additional 24 h or 48 h at 37 °C with 5% CO_2_. Finally, cell viability was assessed using a CCK-8 assay.

## Intracellular ROS detection

ROS production was detected by the cell-permeable dye 2',7'-dichlorofluorescein diacetate (DCFH-DA). RAW 264.7 macrophage cells were inoculated into 24-well plates at a density of 1 × 10^5^ cells per well and incubated at 37 °C, 5% CO_2_ for 24 h. Subsequently, the medium was replaced with 1 mL of medium containing MICP@HG (100 μg/mL), MI, MICP or fresh medium. After 12 h of incubation, the ROS probe was added to each well, and the cells were incubated at 37 °C for 20 min. Finally, the cells were washed three times with serum-free medium. Fluorescence was analyzed using a flow cytometer.

## Macrophage polarization in vitro

RAW 264.7 cells were inoculated into 24-well plates at 1 × 10^5^ cells per well and cultured overnight. After stimulation with 200 ng/mL LPS for 12 h, the cells were rinsed three times with a PBS, and MICP@HG dispersed in DMEM was added for incubation for 24 h. Subsequently, the anti-inflammatory capacity was investigated by flow cytometry using CD86 as an M1 (inflammatory) macrophage marker and CD206 as an M2 (anti-inflammatory) macrophage marker. ELISA assays were performed to quantify the expression of tumor necrosis factor-α (TNF-α), interleukin-6 (IL-6) and transforming growth factor-β (TGF-β).

## Transwell chemotaxis assay

RAW 264.7 cells were inoculated into the upper chamber of a 24 well plate transwell chamber (5 × 10^5^ cells/well), and cultured overnight in medium containing 5% FBS. *E. asburiae* with different treatment (Control, MI, MICP, MICP@HG) was added to the lower chamber and co-cultured for 1 h. The culture medium in the lower chamber was then replaced, and incubation was continued for 24 h. The transwell chamber was fixed with 4% polyformaldehyde for 20 min and washed with PBS three times. After staining with 0.1% crystal violet at room temperature for 20 min, the chamber was washed with PBS twice. The dried transwell chamber was imaged and analyzed using a microscope. ELISA assays were performed to quantify the expression of chemokine (C–C motif) ligand 1 (CCL1) and CCL2.

## Hemolysis assay

Red blood cells (RBCs) were isolated and washed by centrifugation, and diluted with PBS. Then, 0.5 mL of RBC suspension was mixed with 0.5 mL of MICP@HG (0, 100, 200, 300, 400 and 600 μg/mL) in PBS. Deionized (DI) water was used as positive control. The treated suspension was incubated at 37 ℃ for 1 h. After centrifugation, the supernatant was collected and analyzed using a microplate reader [6].

## In vivo biocompatibility of MICP@HG

To evaluate in vivo biocompatibility, BALB/c mice (6-8 weeks) were randomly divided into 5 groups (n = 5) and treated with an antibiotic cocktail (vancomycin (50 μg/mL) + colistin (50 μg/mL)), MI, MICP, MICP@HG (100 μg/mL), or PBS via tail vein injection. After 14 days, serum samples were collected for biochemical analysis, including ALT, ALP, AST, UREA, and CREA, to evaluate liver and kidney function. The heart, liver, spleen, lung, and kidneys were excised for histopathological evaluation by hematoxylin and eosin (H&E) staining.

## In vivo imaging of MICP@HG in mouse bacterial infection models

A muscle infection model was used to evaluate the in vivo targeting ability of the MICP@HG after tail vein injection. Mice (n = 3) were shaved and intramuscularly injected with *E. asburiae* and MRSA (1 × 10^8^ CFU/20 g) on the right thigh (infection site). After 2 h of infection, the mice were injected with MI, MICP, or MICP@HG via the tail vein and imaged at predetermined time points (3, 6, 12, 18, and 24 h) using a small animal living imaging system IVIS (IVIS Lumina III, PerkinElmer, USA).

## MRSA and *E. asburiae*-induced polymicrobial peritonitis infection model

Mice were infected with 0.1 ml of a bacterial suspension (a mixture of MRSA and *E. asburiae*, 5 × 10^7^ CFU for each pathogen) through an intraperitoneal injection. After 0.5 h of infection, the mice were randomly grouped and administered various formulations via tail vein injection, including PBS, an antibiotic cocktail (vancomycin (50μg/mL) + colistin (50μg/mL)), MI, MICP, and MICP@HG (n = 7). The survival rate, clinical scores, body temperature, and body weight of the mice were recorded over time.

Clinical scores of mice were scored according to the following criteria: score 0, no symptoms; score 1, piloerection and huddling; score 2, piloerection, huddling, and diarrhea; score 3, lack of interest in surroundings and severe diarrhea; score 4, decreased movement and listless appearance; and score 5, loss of the self-righting reflex. Mice reaching a score of 5 were humanely euthanized [7].

## CFU detection

In the MRSA and *E. asburiae*-induced polymicrobial peritonitis infection model, mice (n = 6) were sacrificed 24 h after bacterial inoculation and treatment. Peritoneal fluid and major organs (liver, lungs, kidneys) were collected and homogenized in 2 mL of PBS. The CFUs in the peritoneal fluid and infected organs were determined by serial dilution and plate counting.

## Biochemical parameters, inflammatory cytokines, and histopathological analysis

Alanine aminotransferase (ALT), aspartate aminotransferase (AST), creatinine (CREA) and blood urea nitrogen (UREA) were measured on a Hitachiautomatic biochemical analyzer 7180 (Japan). The levels of TNF-α, IL-6 , CRP and TGF-β were measured using ELISA kits. The mouse tissues were fixed in 4% paraformaldehyde for 24 h and then sliced after paraffin embedding. Tissue sections of major organs (lungs, kidneys, liver) were stained with hematoxylin and eosin (H&E) and examined. The injury scores were determined according to a previously established method [8], and the detailed criteria are shown in the following. The sections were prepared using standard procedures, followed by immunohistochemical and immunofluorescence staining.

| Index | 0 | 1 | 2 | 3 | Maximum |
| --- | --- | --- | --- | --- | --- |
| Necrosis | None | Focal piecemeal | Continuous <50% | Continuous >50% | 3 |
| Bleeding | None | <30% | 30-50% | >50% | 3 |
| Infiltration | None | 2 to 3 fold | 3 to 10 fold | >10 fold | 3 |

Kidney injury score parameters

| Renal tubule injury and glomerular shrinkage | Index |
| --- | --- |
| None | 0 |
| <10% | 1 |
| 11–25% | 2 |
| 26–45% | 3 |
| 46–75% | 4 |
| >76% | 5 |

Lung injury score parameters

| Changes in lung tissue structure | Index |
| --- | --- |
| None | 1 |
| Focal interstitial congestion and inflammatory cell infiltration <50% | 2 |
| Diffuse interstitial congestion and inflammatory cell infiltration >50% | 3 |
| Focal consolidation and inflammatory cell infiltration <50% | 4 |
| Diffuse consolidation and inflammatory cell infiltration >50% | 5 |

## In vivo prophylactic effect against bacterial infection

The in vivo prophylactic ability of MICP@HG was verified on the BALB/c mice (6 to 8 weeks) immunized via intraperitoneal injection. On day 3 after immunization, the mice were infected intraperitoneally with *E. asburiae* and MRSA (1 × 10^8^ CFU/20 g). After 24 h of infection, the mice were euthanized and 1 mL of PBS was intraperitoneally injected to collect peritoneal fluid. Subsequently, the liver, lungs, and kidneys were collected and homogenized in PBS. The peritoneal fluid and homogenized suspensions were serially diluted and plated for CFU counting.

## RNA-seq analysis

A 1 mL portion of the bacterial suspension (*E. asburiae*) treated with or without MICP@HG/H_2_O_2_ was collected into a 2 mL Rnase-free tube, followed by centrifugation (4 °C, 6000 rpm, 5 min) to remove the supernatant. Then, the bacteria were quickly frozen in liquid nitrogen for 15 min and stored at −80 °C until further analysis. Finally, RNA sequencing was performed using an Illumina NovaSeq 6000 (Illumina, USA). Transcripts per million reads (TPM) values were used to represent relative gene abundance. Differentially expressed genes (DEGs) were analyzed by DESeq 2. Adjusted p-values (p-adjusted) <0.05 and a fold change >2 were set as the thresholds for the selection of significantly differentially expression genes.

Supporting figure

Figure S1**.** (**a**) TEM image of MSN. (**b**) TEM image of MICP. (**c**) SEM image of MSN. (**d**) SEM image of MICP. (**e**) EDS spectrum of MICP@HG.

Figure S2. (**a**) Absorbance of oxidized TMB with different treatment. (**b**) Lineweaver-Burk plotting for MICP@HG with TMB as a substrate in the presence of H_2_O_2_ at room temperature. (**c**) Michaelis-Menten curves of ROS generation velocities versus the concentration of H_2_O_2_. (**d**) Lineweaver-Burk plotting for MICP@HG with H_2_O_2_ as a substrate in the presence of TMB at room temperature.

Figure S3. (**a**) Oxygen generation under different concentrations of MICP@HG monitored by fluorescence intensity. (**b**) Lineweaver-Burk plotting for MICP@HG with H_2_O_2_ as a substrate. (**c**) GSH consumption under different treatments. (d) Schematic illustration of an in vitro dynamic model simulating the pH-responsive transition from antibacterial to anti-inflammatory activity. (e) Time-dependent changes in intracellular ROS levels in bacteria following treatment. (f) Time-dependent changes in intracellular ROS levels in RAW 264.7 following treatment.

Figure S4. (**a**) Representative fluorescence photos of *E. asburiae*, CREC, MRSA stained with Calcein-AM (green, live bacteria) and PI (red, dead bacteria) in different groups. (**b**-**c**) Quantitative analysis of CREC and MRSA stained with Calcein-AM (green, live bacteria) and PI (red, dead bacteria) in different groups.

Figure S5. Cuproptosis-like death of CREC in vitro. (**a**, **e**) Representative DiBAC_4_(3) staining images and quantitative analysis of CREC after different treatments. (**b**) Extracellular protein concentrations of CREC after different treatments. (**c**) Extracellular nucleic acid concentrations of CREC after different treatments. (**d**, **f**) Representative staining fluorescence photographs and quantitative analysis of ROS (green) of CREC detected by DCFH-DA after different treatments. (**g**) Ratios of GSH/GSSG, (**h**) lipid peroxidation levels, (**i**) pyruvate contents, (**j**) intracellular ATP levels of CREC after different treatments. (**k**) Copper concentration statistics of CREC after different treatments. (**l**) Effect of cuproptosis death inhibitor (EDTA) on anti-CREC activity of MICP@HG. (**m**, **n**) Influence of different treatments on the activity of CREC respiratory chain complexes II and IV.

Figure S6. Cuproptosis-like death of MRSA in vitro. (**a**, **e**) Representative DiBAC_4_(3) staining images and quantitative analysis of MRSA after different treatments. (**b**) Extracellular protein concentrations of MRSA after different treatments. (**c**) Extracellular nucleic acid concentrations of MRSA after different treatments. (**d**, **f**) Representative staining fluorescence photographs and quantitative analysis of ROS (green) of MRSA detected by DCFH-DA after different treatments. (**g**) Ratios of GSH/GSSG, (**h**) lipid peroxidation levels, (**i**) pyruvate contents, (**j**) intracellular ATP levels of MRSA after different treatments. (**k**) Copper concentration statistics of MRSA after different treatments. (**l**) Effect of cuproptosis death inhibitor (EDTA) on anti-MRSA activity of MICP@HG. (**m**, **n**) Influence of different treatments on the activity of MRSA respiratory chain complexes II and IV.

Figure S7. Antibacterial rates of different treatment groups (G1: Control; G2: H_2_O_2_; G3: MICP@HG + H_2_O_2_ + EDTA; G4: MICP@HG + H_2_O_2_ + VC; G5: MICP@HG + H_2_O_2_).

Figure S8. Cytotoxicity of MICP@HG. (**a**) Viability of RAW 264.7 cells treated with different groups for 24h and 48h. (**b**, **c**) Viability of RAW 264.7 cells treated with varying concentrations of MICP@HG for 24h and 48h. (**d**) Viability of HUVEC cells treated with different groups for 24h and 48h. (**e**, **f**) Viability of HUVEC cells treated with varying concentrations of MICP@HG for 24h and 48h (n = 3).

Figure S9. Photographs of bacterial colonies obtained from peritoneal fluid, liver, lung and kidney after different treatments.

Figure S10. (**a**) Detection of the white blood cell (WBC) in the sepsis mice treated with different materials via intravenous injection. (**b**) Detection of neutrophils (Neu) in the sepsis mice treated with different materials via intravenous injection.

Figure S11. **Antioxidant activity, anti-inflammatory and immunomodulatory effects of MICP@HG in vivo.** (**a**) Representative images of liver tissue sections from infected mice under different treatments, showing H&E staining, immunofluorescence staining of TUNEL, Nrf-2, iNOS, and Arg-1, as well as immunohistochemical staining of IL-6 and Ly6G. Quantitative analysis of liver injury scores (**b**) and expression levels of TUNEL (**c**), Nrf-2 (d), iNOS (**e**), Arg-1 (**f**), Ly6G (**g**), and IL-6 (**h**) in liver tissues from infected mice across the treatment groups. (**i**) Radar chart providing a consolidated view of all quantitative metrics.

Figure S12. **Antioxidant activity, anti-inflammatory and immunomodulatory effects of MICP@HG in vivo.** (**a**) Representative images of kidney tissue sections from infected mice under different treatments, showing H&E staining, immunofluorescence staining of TUNEL, Nrf-2, iNOS, and Arg-1, as well as immunohistochemical staining of IL-6 and Ly6G. Quantitative analysis of kidney injury scores (**b**) and expression levels of TUNEL (**c**), Nrf-2 (**d**), iNOS (**e**), Arg-1 (**f**), Ly6G (**g**), and IL-6 (**h**) in kidney tissues from infected mice across the treatment groups. (**i**) Radar chart providing a consolidated view of all quantitative metrics.

Figure S13. **Blood routine analysis of experimental mice after various treatments.** Complete blood cell tests of mice receiving different treatments, including (**a**) white blood cell (WBC), (**b**) lymphocyte (Lym), (**c**) monocyte (Mon), (**d**) neutrophils (Gran), (**e**) red blood cell (RBC), (**f**) hemoglobin (HGB), (**g**) hematocrit (HCT), (**h**) mean corpuscular volume (MCV), (**i**) mean corpuscular hemoglobin (MCH), (**j**) mean corpuscular hemoglobin concentration (MCHC), (**k**) red cell distribution width-standard deviation (RDW), (**l**) platelet count (PLT), (**m**) platelet volume (MPV), (**n**) platelet distribution width (PDW), and (**o**) platelet crit (PCT). The gray area is the normal range for mice.

Figure S14. Blood biochemical analysis of experimental mice after various treatments, including (**a**) urea (UREA), (**b**) alanine aminotransferase (ALT), (**c**) aspartate aminotransferase (AST), (**d**) creatinine (CREA), and (**e**) alkaline phosphatase (ALP). The gray area is the normal range for mice.

Figure S15. (**a**) Hemolysis test of MICP@HG with concentrations from 0 to 300 μg/mL in the mouse blood at 37 °C for 1 h. (**b**) Hemolysis test of MI, MICP, and MICP@HG in the mouse blood at 37 °C for 1 h. PBS and DI water were set as negative and positive controls.

Figure S16. (**a**) In vivo fuorescence images of mice at different days (1, 2, 3, 4, 5, 6, 7, 8, and 9 day) after the intravenous administration of MICP@HG. (**b**) Ex vivo fuorescence images of visceral organs (heart, liver, spleen, lung and kidney) at different days (1, 2, 3, 4, 5, 6, 7, 8, and 9 day) after the intravenous administration of MICP@HG.

Figure S17. Photographs of bacterial colonies obtained from peritoneal fluid, liver, lung and kidney after different treatments.

References

1. Z. Guo, L. Wu, Y. Wang, et al., “Design of Dendritic Large-Pore Mesoporous Silica Nanoparticles with Controlled Structure and Formation Mechanism in Dual-Templating Strategy,” *ACS Applied Materials & Interfaces* 12 (2020): 18823–18832, https://doi.org/10.1021/acsami.0c00596.

2. G. Zhu, Y. Xie, J. Wang, et al., “Multifunctional Copper‐Phenolic Nanopills Achieve Comprehensive Polyamines Depletion to Provoke Enhanced Pyroptosis and Cuproptosis for Cancer Immunotherapy,” *Advanced Materials* 36 (2024): 2409066, https://doi.org/10.1002/adma.202409066.

3. Y. Sun, W. Zhang, Z. Luo, et al., “ZnO‐CuS/F127 Hydrogels with Multienzyme Properties for Implant‐Related Infection Therapy by Inhibiting Bacterial Arginine Biosynthesis and Promoting Tissue Repair,” *Advanced Functional Materials* 35 (2025): 2415778, https://doi.org/10.1002/adfm.202415778.

4. A. N. Schuetz, A. Ferrell, J. A. Hindler, R. Humphries, and A. M. Bobenchik, “Overview of Changes in the Clinical and Laboratory Standards Institute Performance Standards for Antimicrobial Susceptibility Testing: M100 32nd and 33rd Editions,” *Journal of Clinical Microbiology* 63 (2025): e01623-23, https://doi.org/10.1128/jcm.01623-23.

5. Y. Liu, Y. Jia, K. Yang, et al., “Metformin Restores Tetracyclines Susceptibility against Multidrug Resistant Bacteria,” *Advanced Science* 7 (2020): 1902227, https://doi.org/10.1002/advs.201902227.

6. J. Kong, Y. Sun, X. Ge, M. Mao, H. Yu, and Y. Wang, “A Chlorotoxin‐Directed Diselenide‐Bridged Tumor‐Homing Persistent Luminescence Nanoprobes Mediating Inhibition of Oxidative Phosphorylation for Long‐Term Near‐Infrared Imaging and Therapy of Glioblastoma,” *Advanced Functional Materials* 33 (2023): 2209579, https://doi.org/10.1002/adfm.202209579.

7. S. Spence, M. K. Greene, F. Fay, et al., “Targeting Siglecs with a Sialic Acid–Decorated Nanoparticle Abrogates Inflammation,” *Science Translational Medicine* 7 (2015), https://doi.org/10.1126/scitranslmed.aab3459.

8. J. Dawulieti, M. Sun, Y. Zhao, et al., “Treatment of Severe Sepsis with Nanoparticulate Cell-Free DNA Scavengers,” *Science Advances* 6 (2020): eaay7148, https://doi.org/10.1126/sciadv.aay7148.
